# Electromagnetic and heated pulse laser on wave propagation during electrons and holes excitation processes in a rotator semiconductor medium

**DOI:** 10.1038/s41598-025-91584-x

**Published:** 2025-04-15

**Authors:** S. M. Abo-Dahab, Doaa M. Salah, Hanan S. Gafel, A. M. Abd-Alla, M. A. Abdelhafez

**Affiliations:** 1 Department of Mathematics, Faculty of Science, South Valley University, Qena, Egypt; 2 Department of Mathematics, Faculty of Science, Sohag University, Sohag, Egypt; 3 Department of Mathematics and Statistics, College of Science, Taif University, P.O. Box 11099, 21944 Taif, Saudi Arabia

**Keywords:** Magnetic field, Hall current, Laser pulse, Rotation, Semiconductor, Normal mode, Biophysics, Materials science

## Abstract

This study focuses on intricate interplay between electrons and holes and generating a Hall current and its impact on the coupled behavior of thermal, mechanical, and electronic fields in a semiconductor thermoelastic medium. The model indeed considers the motion of microscopic particles (charge carriers such as electrons and holes) by coupling their behaviour with thermal and elastic fields. The process of optical-elastic-thermal-diffusion (OETD) is taken into account when a material is subjected to rotation, time, high electromagnetic fields, and laser pulses. Significant data on the existence of new and enhanced waves in many technological and geophysical applications can be obtained from wave propagation in a thermos-diffusion elastic material. Photoelastic and photoelectronic deformations are accounted for, especially when hall currents impact the semiconductor due to magnetic field pressure. To solve the non-dimensional coupled equations, Lame’s potential and normal mode analysis were employed to simplify the fundamental equations that describe the system in 2-D. Graphical representations of numerically simulated results using MATHEMATICA software highlight the influence of hall current (magnetic field), laser pulse, rotation and time on the results, with a focus on silicon (Si) material. Finding of the current study is that the effect of electromagnetic field on wave propagation heated by pulsed laser during the excitation processes of electrons and holes in a rotator semiconductor medium.

## Introduction

In recent times, there has been a surge of interest in materials science, focusing on understanding their physical characteristics and the behaviour of waves within them. Semiconductors, a class of materials with properties intermediate between electrical conductors (like aluminium) and insulators (such as glass), have garnered significant attention. These semiconductors find widespread applications in modern sectors, including the transistors, electronics industry, solar cells, and electrical circuits. Interestingly, early research on semiconducting materials, like silicon and carbon, treated them as elastic substances, and their behaviour was explored using theories rooted in thermoelasticity. Semiconductors truly play a pivotal role in our daily lives, powering everything from smartphones to household appliances and beyond. Their versatility and impact on technology are remarkable. Recent research in physics shows that charge carriers are particle-free when they are in motion yet nonetheless transport electric charges through the study of semiconductors. The many different types of charge carriers include electrons, ions, and holes, to name just a few. Carriers of charge in semiconductors are holes and electrons. Semiconductors’ outermost atomic layers are populated by free electrons at zero Kelvin. Under these circumstances, electrons or electric currents cannot migrate or alter locations. As a semiconductor’s temperature increases, its internal resistance drops, potentially facilitating the migration of some electrons from the valence band to the conduction band. To be more precise, when electrons are free to flow on a substance’s surface, an electric current is generated. It is known that there is always a vacancy in the valence band when an electron moves to the conduction band. Thus, electrons and holes in semiconductors to be physically close to one other. The motion of free electrons causes electric currents to flow through a semiconductor. When the material is subjected to temperature variations, the pores also transmit electric currents in particular manners. The motion law of microscopic particles such as charge carriers (electrons and holes) in the context of the discussed paper is represented by coupled equations that account for their dynamics in response to thermal, mechanical, and electromagnetic interactions. Here’s how the motion law is generally addressed and its limitations in the paper. These effects require resolving high-frequency oscillations or transient spikes in carrier densities, which are critical for understanding short-time responses. In the beginning, the field of thermoelasticity grappled with the challenge posed by the coupled and uncoupled theories, which did not align well with physical experiments. To address this, Biot^1^ introduced a ground breaking concept: the theory of coupled thermoelasticity. Within this framework, Biot motivated the heat conduction equation and introduced the dynamic theory of thermoelasticity (referred to as the CD theory), drawing upon Fourier’s law of heat. The CD theory delved into the rapid propagation of thermal waves. As research progressed, Lord and Shulman (LS)^2^ introduced another key idea by incorporating a relaxation time into the heat equation. Simultaneously, Green and Lindsay (GL)^3^ extended the heat conduction equation by introducing two relaxation times. Their work culminated in the development of the generalized thermoelasticity theory, which has since been widely explored by various authors^4–6^. The problem presented by Abd-Alla et al.^7^ is the study of the stress, temperature, and magnetic field in an elastic, transversely isotropic cylinder of infinite length with perfectly conducting material placed in a primary constant magnetic field when the cylinder’s curved surface is periodically loaded. Lotfy^8^ investigated the elastic wave motions for a photothermal medium in a dual-phase-lag model with gravitational field and an internal heat source. The semiconductor medium’s density of charge is calculated only as a function of the generated electric current’s time. The issue is examined in relation to thermal memory and laser pulses. Lotfy et al.^9^ analyzed a unique model of the impact of Thomson heating under magnetic field. Hobiny et al.^10^ developed a mathematical model of the Green-Naghdi photo-thermoelastic model using ramp-type heating to investigate photo-thermoelastic waves in a two-dimensional semiconductor material. The variables are derived analytically by using Fourier and Laplace transformations with the eigenvalue scheme. Many authors^11–18^ contribute investigations using some influences on the elastic wave propagations of semiconductor mediums under the photothermal theory. When a strong external magnetic field is applied to a semiconductor material, the Hall current effect cannot be ignored. Magnetic lines cause elastic semiconductors to bend, resulting in free spiralling of electrons and ions and the generation of induced or Hall current^19–23^. Rani et al.^24^ investigated the plane waves at an interface of thermoelastic and magneto-thermoelastic media. The physical properties of light fields at the subwavelength scale have emerged as extensively pursued objectives in nano-optics, photonics, and plasmonics^25–34^.

**Fig. 1:**
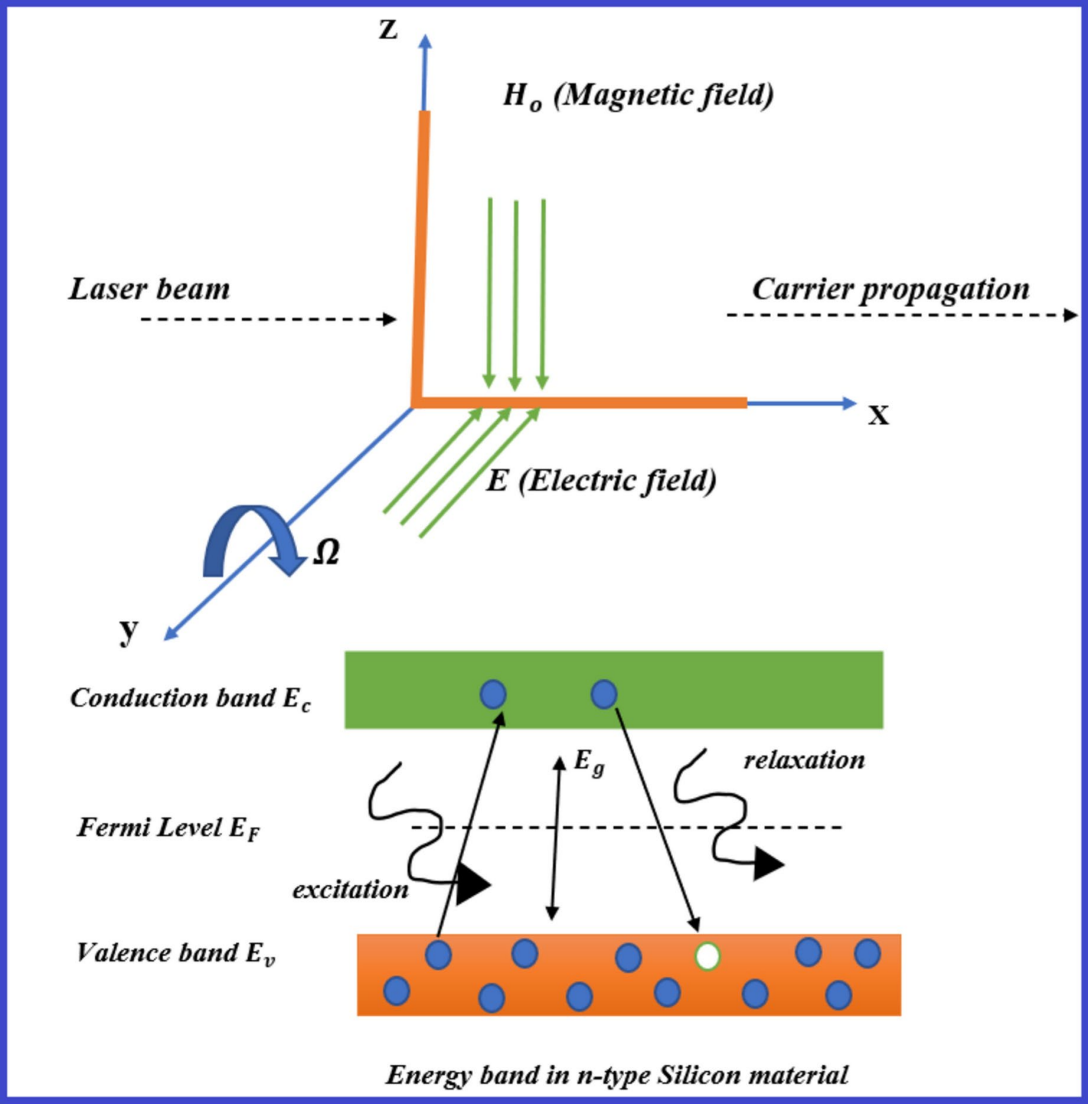
Geometry of the problem.

This research looks at how a powerful magnetic field affects the Hall current as a consequence of electron–hole interactions. The effects of laser pulses on photo-generated charges in semiconductor media, including their optical, elastic, and thermal properties, are taken into account. These dynamics are governed by mechanisms such as drift, diffusion, and recombination-generation processes, which are much faster compared to other coupled fields. Thermal and elastic wave propagation operates on much longer timescales, often ranging from microseconds to milliseconds or more, depending on the material properties and the size of the system. The inertia and slower propagation speeds of mechanical and thermal waves make their dynamics distinctly slower than those of charge carriers. A continuum approach where the collective behavior of electrons and holes is described by field variables, such as the plasma density and hole charge carrier concentration is assumed. These quantities are influenced by external thermal gradients, magnetic fields, and optical excitation, effectively coupling their dynamics with thermoelastic and photothermal processes. The governing equations incorporate these effects but simplify the microscopic details of charge carrier motion into averaged quantities. The interaction between the thermal, elastic, and electronic fields is achieved through coupled equations, but these equations primarily treat the charge carriers as part of the macroscopic continuum, not as discrete particles. Also, this study investigates how a strong magnetic field affects the Hall current caused by electron–hole interactions in semiconductor materials. Furthermore, taking into consideration their optical, elastic, and thermal characteristics, the impacts of rotation and laser pulses on photo-generated charges are taken into account. The fundamental equation governing electronics and thermoelastic deformation is introduced in (2D) when thermo-diffusive processes transfer mass and heat. Analytical Solutions (Principal fields) are obtained through analytical methods as Lame’s potential and normal mode analysis. Numerical Simulations explore wave propagation and fundamental physical characteristics in semiconductors using silicon’s physical constants as an example. The paper does not explicitly address the time-scale disparity between the rapid dynamics of electrons/holes and the slower thermal-elastic waves. This oversight can lead to inaccuracies in modeling the coupled behavior since the assumption of simultaneous dynamics for all fields is not physically realistic. The continuum model treats all physical quantities (temperature, displacement, charge carrier density) as if they evolve on similar timescales. This simplification may neglect transient phenomena unique to the fast-moving charge carriers. The dynamic behavior of electrons and holes, such as their transient response to optical excitation or electromagnetic fields, is not adequately modeled. The findings are visually presented and thoroughly explained. The results are compared to several hypotheses and in the presence and absence of magnetic fields, rotation, and laser pulses. The obtained results depict that the influences of rotation, laser pulse, magnetic field, time and diffusion are all significant (Fig. 1).

## Basic equations and formulation

Consider a 2-D semiconductor medium in an equilibrium state that is isotropic, thermoelastic, and homogeneous. Let $(u, w) (x, t), T(x, t), N(x, t),$ and $H (x, t)$ represent the displacement vectors, temperature change, electron charge carrier concentration, and hole charge carrier concentration, respectively. The main equations, describing the interaction between thermal, elastic, hole, and electron–hole charge distributions, can be formulated in the plane $x-z$ under the influences of Electromagnetic field, Laser pulse, Rotation. These equations constitute a mathematical-physical model that captures the phenomenon of electron–hole interaction induced by laser pulses and an internal heat source in semiconductors in the absence of body forces and in the framework of the photo-electronic/thermoelastic deformation processes, following the linear theory of thermoelasticity.

It is possible to get three models in photo-thermoelasticity theory by varying thermal and elastic relaxation durations. The parameter $\tau_{\theta }$ represents the first phase of lag, whereas the value $\tau_{q}$ represents the second. However, this inquiry has three models:

1. The dual phase lag (DPL) model can be obtained when $0 \le \tau_{\theta } < \tau_{q}$.
2. The Lord and Shulman (LS) model can be obtained when $\tau_{\theta } = 0 x$,$0 < \tau_{q}$.
3. The coupled thermoelasticity (CT) model can be obtained when $\tau_{\theta } = \tau_{q} = 0$.

Under the influence of laser pulses, the temperature, displacement, carrier density, and hole charge carrier field (electronic, thermal, and elastic characteristics) were connected by the heat conduction equation that is

1 $$\begin{gathered} k\left( {1 + \tau_{\theta } \frac{\partial }{\partial t}} \right)\nabla^{2} T + m_{nq} \nabla^{2} N + m_{hq} \nabla^{2} H - \rho \left( {a_{1}^{n} \frac{\partial N}{{\partial t}} + a_{1}^{h} \frac{\partial H}{{\partial t}}} \right) - \left( {1 + \tau_{q} \frac{\partial }{\partial t}} \right)\left[ {\rho c_{e} \frac{\partial T}{{\partial t}}} \right. \hfill \\ \left. { + \rho T_{0} \alpha_{n} \frac{\partial N}{{\partial t}} + \rho T_{0} \alpha_{h} \frac{\partial H}{{\partial t}} + \gamma T_{0} \frac{\partial }{\partial t}\left( {\frac{\partial u}{{\partial x}} + \frac{\partial w}{{\partial z}}} \right)} \right] - \left( {\frac{{\rho a_{1}^{n} }}{{t^{n} }}N + \frac{{\rho a_{1}^{h} }}{{t^{h} }}H} \right) = \left( {1 + \tau_{q} \frac{\partial }{\partial t}} \right)P\delta e^{{ - (I_{0} t + \delta x)}} \hfill \\ \end{gathered}$$

The (thermal/plasma/elastic) equations are

2 $$m_{qn} \nabla^{2} T + D_{n} \rho \nabla^{2} N - \rho \left( {1 - a_{2}^{n} T_{o} \alpha_{n} + \tau^{n} \frac{\partial }{\partial t}} \right)\frac{\partial N}{{\partial t}} - a_{2}^{n} \left[ {\rho c_{e} \frac{\partial T}{{\partial t}} + \rho T_{o} \alpha_{h} \frac{\partial H}{{\partial t}} + \gamma T_{o} \frac{\partial }{\partial t}\left( {\frac{\partial u}{{\partial x}} + \frac{\partial w}{{\partial z}}} \right)} \right] + \frac{\rho }{{t_{1}^{n} }}\left( {1 + \tau^{n} \frac{\partial }{\partial t}} \right)N = 0$$

3 $$\begin{gathered} m_{qh} \nabla^{2} T + D_{h} \rho \nabla^{2} H - \rho \left( {1 - \alpha_{2}^{h} T_{0} \alpha_{h} + t^{h} \frac{\partial }{\partial t}} \right)\frac{\partial H}{{\partial t}} - \alpha_{2}^{h} \left[ {\rho c_{e} \frac{\partial T}{{\partial t}} + \rho T_{0} \alpha_{n} \frac{\partial N}{{\partial t}}} \right. \hfill \\ \left. { + \gamma T_{0} \frac{\partial }{\partial t}\left( {\frac{\partial u}{{\partial x}} + \frac{\partial w}{{\partial z}}} \right)} \right] + \frac{\rho }{{t_{1}^{h} }}\left( {1 + t^{h} \frac{\partial }{\partial t}} \right)H = 0, \hfill \\ \end{gathered}$$

The Hall current phenomenon, which illustrates the effects of electrical current flowing through a semiconductor media subjected to a strong magnetic field, might be used to formulate the corresponding equation of motion

4 $$\begin{gathered} {\uprho }\left( {\frac{{\partial^{2} {\text{u}}}}{{\partial {\text{t}}^{2} }} - {\Omega }^{2} {\text{u}} + 2{\Omega }\frac{{\partial {\text{w}}}}{{\partial {\text{t}}}}} \right) = \left( {{\uplambda } + 2{\upmu } + {\upmu }_{{\text{e}}} {\text{\rm H}}_{^\circ }^{2} } \right)\frac{{\partial^{2} {\text{u}}}}{{\partial {\text{x}}^{2} }} + \left( {{\uplambda } + {\upmu } + {\upmu }_{{\text{e}}} {\text{\rm H}}_{^\circ }^{2} } \right)\frac{{\partial^{2} {\text{w}}}}{{\partial {\text{x}}\partial {\text{z}}}} \hfill \\ + {\upmu }\frac{{\partial^{2} {\text{u}}}}{{\partial {\text{z}}^{2} }} - \gamma \left( {1 + \tau_{\theta } \frac{\partial }{\partial t}} \right)\frac{\partial T}{{\partial x}} - \delta_{n} \frac{\partial N}{{\partial x}} - \delta_{h} \frac{\partial H}{{\partial x}} - \left( {\frac{{\sigma_{o} {\upmu }_{{\text{e}}} H_{o}^{2} }}{{1 + m^{2} }}} \right)\frac{{\partial^{2} {\text{u}}}}{{\partial {\text{t}}^{2} }}, \hfill \\ \end{gathered}$$

5 $$\begin{gathered} {\uprho }\left( {\frac{{\partial^{2} {\text{w}}}}{{\partial {\text{t}}^{2} }} - {\Omega }^{2} {\text{w}} + 2{\Omega }\frac{{\partial {\text{u}}}}{{\partial {\text{t}}}}} \right) = \left( {{\uplambda } + 2{\upmu } + {\upmu }_{{\text{e}}} {\text{\rm H}}_{^\circ }^{2} } \right)\frac{{\partial^{2} {\text{w}}}}{{\partial {\text{z}}^{2} }} + \left( {{\uplambda } + {\upmu } + {\upmu }_{{\text{e}}} {\text{\rm H}}_{^\circ }^{2} } \right)\frac{{\partial^{2} {\text{u}}}}{{\partial {\text{x}}\partial {\text{z}}}} \hfill \\ + {\upmu }\frac{{\partial^{2} {\text{w}}}}{{\partial {\text{x}}^{2} }} - \gamma \left( {1 + \tau_{\theta } \frac{\partial }{\partial t}} \right)\frac{\partial T}{{\partial z}} - \delta_{n} \frac{\partial N}{{\partial z}} - \delta_{h} \frac{\partial H}{{\partial z}} - \left( {\frac{{\sigma_{o} {\upmu }_{{\text{e}}} H_{o}^{2} }}{{1 + m^{2} }}} \right)\frac{{\partial^{2} {\text{w}}}}{{\partial {\text{t}}^{2} }}, \hfill \\ \end{gathered}$$

The stress–strain– heat plasma equations for the field of electron/hole carriers are

6 $${\upsigma }_{{{\text{xx}}}} = \left( {{\uplambda } + 2{\upmu }} \right)\frac{{\partial {\text{u}}}}{{\partial {\text{x}}}} + {\uplambda }\frac{{\partial {\text{w}}}}{{\partial {\text{z}}}} + \delta_{n} N + \delta_{h} H - {\upgamma }\left( {1 + \tau_{\theta } \frac{\partial }{\partial t}} \right)T,$$

7 $${\upsigma }_{{{\text{zz}}}} = \left( {{\uplambda } + 2{\upmu }} \right)\frac{{\partial {\text{w}}}}{{\partial {\text{z}}}} + {\uplambda }\frac{{\partial {\text{u}}}}{{\partial {\text{x}}}} + \delta_{n} N + \delta_{h} H - \gamma \left( {1 + \tau_{\theta } \frac{\partial }{\partial t}} \right)T,$$

8 $$\upsigma _{{{\text{yy}}}} = \left( {\uplambda + {\text{p}}} \right)\left( {\frac{{\partial {\text{u}}}}{{\partial {\text{x}}}} + \frac{{\partial {\text{w}}}}{{\partial {\text{z}}}}} \right) - \upgamma {\text{T}},$$

9 $$\tau_{{{\text{xz}}}} = {\upmu }\left( {\frac{{\partial {\text{u}}}}{{\partial {\text{z}}}} + \frac{{\partial {\text{w}}}}{{\partial {\text{x}}}}} \right)$$

The dimensionless variables listed below can be used to improve the transformation of the main quantities in the dimensionless case:

10 $$\begin{gathered} \left( {\acute{x},\acute{z},\acute{u},\acute{w}} \right) = \frac{{\omega ^{*} }}{{c_{T} }}~\left( {x,z,~u,w} \right),k^{\prime} = \frac{k}{{\rho c_{e} }}~,N^{\prime} = \frac{{\delta _{n} N}}{{\left( {\lambda + 2\mu } \right)}},T^{\prime} = \frac{{\gamma T}}{{\left( {\lambda + 2\mu } \right)}},\sigma _{{ij}}^{'} = \frac{{\sigma _{{ij}} }}{{\lambda + 2\mu ~}}, \hfill \\ \delta ^{\prime} = \frac{{\delta k}}{{\rho c_{e} c_{T} }},I^{\prime}_{o} = \frac{{I_{o} k}}{{\rho c_{e} c_{T}^{2} }}\left( {t^{\prime},~\tau _{\theta }^{'} ,~\tau _{q}^{'} ,t^{{n'}} ,t^{{h'}} ,t_{1}^{{n^{\prime}}} ,t_{1}^{{h^{\prime}}} } \right) = \omega ^{*} \left( {t,~\tau _{\theta } ,\tau _{q} ,t^{n} ,t^{h} ,t_{1}^{n} ,t_{1}^{h} } \right), \hfill \\ \left( {\delta ^{\prime}_{n} ,\delta ^{\prime}_{h} } \right) = \frac{{\left( {\delta _{n} n_{o} ,\delta _{h} h_{o} } \right)}}{{\gamma T_{o} }},~H^{\prime} = \frac{{\delta _{n} H}}{{\lambda + 2\mu }},c_{T}^{2} = \frac{{\lambda + 2\mu }}{\rho },\omega ^{*} = \frac{{c_{e} \left( {\lambda + 2\mu } \right)}}{k}, \acute{\Omega} = \frac{\Omega }{{\omega ^{*} }} \hfill \\ \end{gathered}$$

To simplify the presentation of the governing Eqs. (9)–(11) with Eq. (10) dashes are removed. Consequently, it generates

11 $$\begin{gathered} \left( {1 + \tau_{\theta } \frac{\partial }{\partial t}} \right)\nabla^{2} T + \alpha_{1} \nabla^{2} N + \alpha_{2} \nabla^{2} H - \alpha_{3} \left( {a_{1}^{n} \frac{\partial N}{{\partial t}} + a_{1}^{h} \frac{\partial H}{{\partial t}}} \right) - \left( {1 + \tau_{q} \frac{\partial }{\partial t}} \right)\left[ {\alpha_{4} \frac{\partial T}{{\partial t}} + \alpha_{5} \frac{\partial N}{{\partial t}}} \right. \hfill \\ \left. { + \alpha_{6} \frac{\partial H}{{\partial t}} + \alpha_{7} \frac{\partial }{\partial t}\left( {\frac{\partial u}{{\partial x}} + \frac{\partial w}{{\partial z}}} \right)} \right] - \left[ {\alpha_{8} N + \alpha_{9} H} \right] = \left( {1 + \tau_{q} \frac{\partial }{\partial t}} \right){\Gamma }_{1} e^{{ - a\left( {I_{o} t + \delta x} \right)}} \hfill \\ \end{gathered}$$

12 $$\nabla^{2} T + \alpha_{10} \nabla^{2} N - \alpha_{11} \left( {1 - a_{2}^{n} T_{o} \alpha_{n} + \tau^{n} \frac{\partial }{\partial t}} \right)\frac{\partial N}{{\partial t}} - a_{2}^{n} \left[ {\alpha_{12} \frac{\partial T}{{\partial t}} + \alpha_{13} \frac{\partial H}{{\partial t}} + \alpha_{14} \frac{\partial }{\partial t}\left( {\frac{\partial u}{{\partial x}} + \frac{\partial w}{{\partial z}}} \right)} \right] + \alpha_{15} \left( {1 + \tau^{n} \frac{\partial }{\partial t}} \right)N = 0$$

13 $$\nabla^{2} T + \alpha_{16} \nabla^{2} H - \alpha_{17} \left( {1 - a_{2}^{h} T_{o} \alpha_{h} + t^{h} \frac{\partial }{\partial t}} \right)\frac{\partial H}{{\partial t}} - \left[ {\alpha_{18} \frac{\partial T}{{\partial t}} + \alpha_{19} \frac{\partial N}{{\partial t}} + \alpha_{20} \frac{\partial }{\partial t}\left( {\frac{\partial u}{{\partial x}} + \frac{\partial w}{{\partial z}}} \right)} \right] + \alpha_{21} \left( {1 + t^{h} \frac{\partial }{\partial t}} \right)H = 0$$

14 $$\left( {\frac{{\partial^{2} {\text{u}}}}{{\partial {\text{t}}^{2} }} - {\Omega }^{2} {\text{u}} + 2{\Omega }\frac{{\partial {\text{w}}}}{{\partial {\text{t}}}}} \right) = \alpha_{22} \frac{{\partial^{2} {\text{u}}}}{{\partial {\text{x}}^{2} }} + \alpha_{23} \frac{{\partial^{2} {\text{w}}}}{{\partial {\text{x}}\partial {\text{z}}}} + \alpha_{24} \frac{{\partial^{2} {\text{u}}}}{{\partial {\text{z}}^{2} }} - \alpha_{25} \left( {1 + \tau_{\theta } \frac{\partial }{\partial t}} \right)\frac{\partial T}{{\partial x}} - \alpha_{26} \frac{\partial N}{{\partial x}} - \alpha_{27} \frac{\partial H}{{\partial x}} - \alpha_{28} \frac{\partial u}{{\partial t}},$$

15 $$\left( {\frac{{\partial^{2} {\text{w}}}}{{\partial {\text{t}}^{2} }} - {\Omega }^{2} {\text{w}} + 2{\Omega }\frac{{\partial {\text{u}}}}{{\partial {\text{t}}}}} \right) = \alpha_{29} \frac{{\partial^{2} {\text{w}}}}{{\partial {\text{z}}^{2} }} + \alpha_{30} \frac{{\partial^{2} {\text{u}}}}{{\partial {\text{x}}\partial {\text{z}}}} + \alpha_{31} \frac{{\partial^{2} {\text{w}}}}{{\partial {\text{x}}^{2} }} - \alpha_{32} \left( {1 + \tau_{\theta } \frac{\partial }{\partial t}} \right)\frac{\partial T}{{\partial z}} - \alpha_{33} \frac{\partial N}{{\partial z}} - \alpha_{34} \frac{\partial H}{{\partial z}} - \alpha_{35 } \frac{\partial w}{{\partial t}},$$

16 $$\sigma_{xx} = \frac{\partial u}{{\partial x}} + \alpha_{36} \frac{\partial w}{{\partial z}} + \alpha_{37} N + \alpha_{38} H - \left( {1 + \tau_{\theta } \frac{\partial }{\partial t}} \right)T$$

17 $$\sigma_{zz} = \frac{\partial w}{{\partial z}} + \alpha_{36} \frac{\partial u}{{\partial x}} + \alpha_{37} N + \alpha_{38} H - \left( {1 + \tau_{\theta } \frac{\partial }{\partial t}} \right)T$$

18 $$\tau_{xz} = \frac{\mu }{\lambda + 2\mu }\left( {\frac{\partial u}{{\partial z}} + \frac{\partial w}{{\partial x}}} \right).$$

where $$\alpha_{1} = \frac{{m_{nq} {\upgamma }}}{{\delta_{n} \rho c_{e} k}}$$, $$\alpha_{2} = \frac{{m_{hq} {\upgamma }}}{{\delta_{n} \rho c_{e} k}}$$, $$\alpha_{3} = \frac{{{\upgamma }c_{T}^{2} }}{{\delta_{n} c_{e} k\omega^{*} }}$$, $$\alpha_{4} = \frac{{c_{T}^{2} }}{{k\omega^{*} }}$$, $$\alpha_{5} = \frac{{T_{o} \alpha_{n} {\upgamma }c_{T}^{2} }}{{\delta_{n} c_{e} k\omega^{*} }}$$, $$\alpha_{6} = \frac{{T_{o} \alpha_{h} {\upgamma }c_{T}^{2} }}{{\delta_{n} c_{e} k\omega^{*} }},$$ $$\alpha_{7} = \frac{{{\upgamma }^{2} T_{o} c_{T}^{2} }}{{\delta_{n} c_{e} k\omega^{*} \left( {{\uplambda } + 2{\upmu }} \right)}}$$, $$\alpha_{8} = \frac{{{\upgamma }c_{T}^{2} }}{{\delta_{n} c_{e} k\omega^{*} }}$$, $$\alpha_{9} = \frac{{{\upgamma }c_{T}^{2} }}{{\delta_{n} c_{e} k\omega^{*} }},$$ $${\text{a}} = \frac{{\rho c_{e} c_{T}^{2} }}{{k\omega^{*} }},$$ $$\Gamma_{1} = \frac{{p\delta \gamma c_{T}^{3} }}{{k^{2} \omega^{*2} \left( {\lambda + 2\mu } \right)}}$$, $$a = \frac{{\rho c_{e} c_{T}^{2} }}{{k\omega^{*} }}$$, $$\alpha_{10} = \frac{{D_{n} \rho {\upgamma }}}{{\delta_{n} m_{qn} }}$$, $$\alpha_{11} = \frac{{\rho {\upgamma }c_{T}^{2} }}{{\delta_{n} m_{qn} \omega^{*} }}$$, $$\alpha_{12} = \frac{{\rho c_{e} c_{T}^{2} }}{{m_{qn} \omega^{*} }}$$, $$\alpha_{13} = \frac{{\rho T_{o} \alpha_{h} {\upgamma }c_{T}^{2} }}{{\delta_{n} m_{qn} \omega^{*} }}$$, $$\alpha_{14} = \frac{{T_{o} {\upgamma }^{2} c_{T}^{2} }}{{m_{qn} \omega^{*} \left( {{\uplambda } + 2{\upmu }} \right)}}$$, $$\alpha_{15} = \frac{{\rho {\upgamma }c_{T}^{2} }}{{t_{1}^{n} \delta_{n} m_{qn} \omega^{*} }},$$ $$\alpha_{16} = \frac{{D_{h} \rho {\upgamma }}}{{\delta_{n} m_{qh} }}$$, $$\alpha_{17} = \frac{{\rho {\upgamma }c_{T}^{2} }}{{\delta_{n} m_{qh} \omega^{*} }}$$, $$\alpha_{18} = \frac{{a_{2}^{h} \rho c_{e} c_{T}^{2} }}{{m_{qh} \omega^{*} }}$$, $$\alpha_{19} = \frac{{a_{2}^{h} \rho T_{o} \alpha_{n} c_{T}^{2} {\upgamma }}}{{m_{qh} \omega^{*} }}$$, $$\alpha_{20} = \frac{{T_{o} {\upgamma }^{2} c_{T}^{2} }}{{m_{qh} \omega^{*} \left( {{\uplambda } + 2{\upmu }} \right)}}$$, $$\alpha_{21} = \frac{{\rho c_{T}^{2} {\upgamma }}}{{t_{1}^{h} m_{qh} \omega^{*} \delta_{n} }},$$ $$\alpha_{22} = \frac{{{\uplambda } + 2{\upmu } + {\upmu }_{{\text{e}}} {\text{\rm H}}_{^\circ }^{2} }}{{\rho c_{T} \omega^{*} }}$$, $$\alpha_{23} = \frac{{{\uplambda } + {\upmu } + {\upmu }_{{\text{e}}} {\text{\rm H}}_{^\circ }^{2} }}{{\rho c_{T}^{2} }}$$, $$\alpha_{24} = \frac{{\upmu }}{{\rho c_{T} \omega^{*} }}$$, $$\alpha_{25} = \frac{{{\uplambda } + 2{\upmu }}}{{\rho c_{T}^{2} }}$$, $$\alpha_{26} = \frac{{{\upgamma }T_{o} \left( {{\uplambda } + 2{\upmu }} \right)}}{{n_{o} \rho c_{T}^{2} }},$$ $$\alpha_{27} = \frac{{{\upgamma }T_{o} \delta_{h} \left( {{\uplambda } + 2{\upmu }} \right)}}{{\delta_{n} h_{o} \rho c_{T}^{2} }},$$ $$\alpha_{28} = \frac{{{\upmu }_{{\text{e}}} H_{o}^{2} \sigma_{o} }}{{\rho \left( {1 + m^{2} } \right)}}$$, $$\alpha_{29} = \frac{{{\uplambda } + 2{\upmu } + {\upmu }_{{\text{e}}} {\text{\rm H}}_{^\circ }^{2} }}{\rho }$$, $$\alpha_{30} = \frac{{{\uplambda } + {\upmu } + {\upmu }_{{\text{e}}} {\text{\rm H}}_{^\circ }^{2} }}{{\rho c_{T}^{2} }}$$, $$\alpha_{31} = \frac{{\upmu }}{{\rho c_{T}^{2} }}$$, $$\alpha_{32} = \frac{{{\uplambda } + 2{\upmu }}}{{\rho c_{T}^{2} }}$$, $$\alpha_{33} = \frac{{\delta_{n} {\upgamma }T_{o} \left( {{\uplambda } + 2{\upmu }} \right)}}{{n_{o} \rho c_{T}^{2} \delta_{h} }}$$, $$\alpha_{34} = \frac{{{\upgamma }T_{o} \delta_{h} \left( {{\uplambda } + 2{\upmu }} \right)}}{{\delta_{n} h_{o} \rho c_{T}^{2} }}$$, $$\alpha_{35} = \frac{{{\upmu }_{{\text{e}}} H_{o}^{2} \sigma_{o} }}{{\rho \left( {1 + m^{2} } \right)}}$$, $$\alpha_{36} = \frac{\lambda }{{\lambda + 2{\upmu }}}$$, $$\alpha_{37} = \frac{{\gamma T_{o} }}{{n_{o} }}$$, $$\alpha_{38} = \frac{{\gamma T_{o} \delta_{h} }}{{h_{o} \delta_{n} }}$$.

## Solution of the problem

This section presents the application of Lame’s potential and normal mode methods to solve the issue with accuracy and without assuming any constraints on the field variables that are included in the governing equations. Presenting the displacement potentials $\phi (x,z,t)$ and $\psi (x,z,t)$ which are connected to the displacement components by the following relations:

19 $$u = \frac{\partial \phi }{{\partial x}} - \frac{\partial \psi }{{\partial z}}, w = \frac{\partial \phi }{{\partial z}} + \frac{\partial \psi }{{\partial x}}$$

The following normal modes can be used to investigate the physical variable solutions:

20 $$\left( {u,w,\phi ,\psi ,T,N,\sigma_{ij} } \right)\left( {x,z,t} \right) = \left( {u^{*} ,w^{*} ,\phi^{*} ,\psi^{*} ,T^{*} ,N^{*} ,\sigma_{ij}^{*} } \right)e^{{\left( {\omega t + ibz} \right)}}$$

where the angular frequency, the imaginary number, and the wave number in the $z$-direction are denoted by the letters $\omega , i, \;{\text{and}}\;b$.

Employing Eqs. (19) and (20), Eqs. (11–15) become, respectively:

21 $$(\beta_{18} D^{2} - \beta_{20} D - \beta_{21} )T^{*} + (\alpha_{1} D^{2} - \beta_{22} )N^{*} + (\alpha_{2} D^{2} - \beta_{23} )H^{*} - (\beta_{19} D^{2} - \beta_{24} )\phi^{*} = R_{H} e^{{ - \left( {\delta x + ibz} \right)}} ,$$

22 $$(D^{2} - \beta_{6} )T^{*} + (\alpha_{10} D^{2} - \beta_{7} )N^{*} + \beta_{9} H^{*} + (\beta_{5} D^{2} - \beta_{8} )\phi^{*} = 0,$$

23 $$(D^{2} - \beta_{11} )T^{*} - \beta_{12} N^{*} + (\alpha_{16} D^{2} - \beta_{13} )H^{*} + (\beta_{10} D^{2} - \beta_{14} )\phi^{*} = 0,$$

24 $$\beta_{15} T^{*} + \alpha_{26} N^{*} + \alpha_{27} H^{*} - (\alpha_{22} D^{2} + \beta_{16} )\phi^{*} = 0,$$

25 $$(\alpha_{31} D^{2} - \beta_{17} )\psi^{*} = 0,$$

where $$D^{2} = \frac{{d^{2} }}{{dx^{2} }}$$, $$\beta_{1} = 1 + \alpha_{28}$$, $$\beta_{2} = \alpha_{23} + \alpha_{24}$$, $$\beta_{3} = 1 + \alpha_{35}$$, $$\beta_{4} = \alpha_{29} + \alpha_{30}$$, $$\beta_{5} = \omega \alpha_{14},$$ $$\beta_{6} = b^{2} + a_{2}^{n} \omega \alpha_{12}$$, $$\beta_{7} = b^{2} \alpha_{10} + \alpha_{11} \left( {1 - a_{2}^{n} T_{o} \alpha_{n} + \tau^{n} \omega - \alpha_{15} \left( {1 + \tau^{n} \omega } \right)} \right)$$, $$\beta_{8} = b^{2} \omega \alpha_{14},$$ $$\beta_{9} = \omega \alpha_{19},$$ $$\beta_{10} = \omega \alpha_{2}$$, $$\beta_{11} = b^{2} + \omega \alpha_{18}$$, $$\beta_{12} = \omega \alpha_{19}$$, $$\beta_{13} = b^{2} \alpha_{16} + \omega \alpha_{17} - \alpha_{21} \left( {1 + t^{h} \omega } \right),$$ $$\beta_{14} = b^{2} \omega,$$ $$\beta_{15} = \alpha_{25} \left( {1 + \tau_{\theta } } \right)$$, $$\beta_{16} = {\Omega }^{2} - \beta_{1} \omega^{2} - b^{2} \beta_{2},$$ $$\beta_{17} = - {\Omega }^{2} + \beta_{3} \omega^{2} + b^{2} \beta_{4}$$, $$\beta_{18} = \left( {1 + \tau_{\theta } \omega } \right),$$ $$\beta_{19} = \omega \alpha_{7}$$, $$\beta_{20} = \alpha_{4} \left( {1 + \tau_{q} \omega } \right)$$, $$\beta_{21} = {\text{b}}^{2} \left( {1 + \tau_{\theta } \omega } \right)$$, $$\beta_{22} = \left( {{\text{b}}^{2} \alpha_{1} + a_{1}^{n} \omega \alpha_{3} + \left( {1 + \tau_{q} \omega } \right)\omega \alpha_{5} + \alpha_{8} } \right)$$, $$\beta_{23} = \left( {{\text{b}}^{2} \alpha_{2} + a_{1}^{h} \omega \alpha_{3} + \left( {1 + \tau_{q} \omega } \right)\omega \alpha_{6} + \alpha_{9} } \right)$$, $$\beta_{24} = {\text{b}}^{2} \omega \alpha_{7} , R_{H} = \left( {1 + \tau_{q} \omega } \right){\Gamma }_{1} e^{{ - \left( {I_{o} t + \omega t} \right)}}$$.

Eliminating $T^{*} \left( x \right),{ }N^{*} \left( x \right),H^{*} \left( x \right)$, and $\phi^{*} \left( x \right)$ from Eqs. (22–24) yields the following eighth order.

26 $$[D^{8} + A_{11} D^{6} + A_{22} D^{4} + A_{33} D^{2} + A_{44} ]\left\{ {T^{*} \left( x \right),{ }N^{*} \left( x \right),H^{*} \left( x \right),{ }\phi^{*} \left( x \right)} \right\} = 0$$

The characteristic equation of Eq. (26) is

27 $$k^{8} + A_{11} k^{6} + A_{22} k^{4} + A_{33} k^{2} + A_{44} = 0$$

where, the definitions for the coefficients $A_{11} ,\,A_{22} ,\,\,A_{33} ,\,\,A_{44}$ involved are detailed in Appendix 1,

$$\xi = \alpha_{22} \left( {\alpha_{1} \alpha_{16} + \alpha_{10} \left( {\alpha_{2} - \alpha_{16} \beta_{18} } \right)} \right)$$,

$k_{n} ,$ $n = 1, 2, 3, 4, 5, 6, 7, 8$ are the all roots for this equation.

The general solutions of Eqs. (22)–(25), bound as *x* → ∞, are given by:

28 $$T^{*} = \mathop \sum \limits_{n = 1}^{4} L_{n} e^{{ - k_{n} x}}$$

29 $$\phi^{*} = \mathop \sum \limits_{n = 1}^{4} H_{1n} L_{n} e^{{ - k_{n} x}}$$

30 $$H^{*} = \mathop \sum \limits_{n = 1}^{4} H_{2n} L_{n} e^{{ - k_{n} x}}$$

31 $$N^{*} = \mathop \sum \limits_{n = 1}^{4} H_{3n} L_{n} e^{{ - k_{n} x}}$$

32 $$\psi^{*} = L_{5} e^{{ - \sqrt {\frac{{{\upbeta }_{17} }}{{\alpha_{31} }}} x}}$$

The displacement components may be calculated in the following manner by using Eqs. (19), (20), (29) and (32):

33 $$u^{*} = - \left\{ {\mathop \sum \limits_{n = 1}^{4} k_{n} H_{1n} L_{n} e^{{ - k_{n} x}} + ibL_{5} e^{{ - \sqrt {\frac{{{\upbeta }_{17} }}{{\alpha_{31} }}} x}} } \right\}$$

34 $$w^{*} = \left\{ {\mathop \sum \limits_{n = 1}^{4} ibH_{1n} L_{n} e^{{ - k_{n} x}} - \sqrt {\frac{{{\upbeta }_{17} }}{{\alpha_{31} }}} L_{5} e^{{ - \sqrt {\frac{{{\upbeta }_{17} }}{{\alpha_{31} }}} x}} } \right\}$$

By using Eqs. (16)–(18), (19), and (28)–(32), the stress components may be determined

35 $$\sigma_{xx}^{*} = \left\{ {\mathop \sum \limits_{n = 1}^{4} R_{s} L_{n} e^{{ - k_{n} x}} - R_{t} L_{5} e^{{ - \sqrt {\frac{{{\upbeta }_{17} }}{{\alpha_{31} }}} x}} } \right\}$$

36 $$\sigma_{zz}^{*} = \left\{ {\mathop \sum \limits_{n = 1}^{4} R_{w} L_{n} e^{{ - k_{n} x}} - R_{b} L_{5} e^{{ - \sqrt {\frac{{{\upbeta }_{17} }}{{\alpha_{31} }}} x}} } \right\}$$

37 $$\tau_{xz}^{*} = \frac{ - \mu }{{\lambda + 2\mu }}\left\{ {\mathop \sum \limits_{n = 1}^{4} 2ibk_{n} H_{1n} L_{n} e^{{ - k_{n} x}} - k_{13} L_{5} e^{{ - \sqrt {\frac{{{\upbeta }_{17} }}{{\alpha_{31} }}} x}} } \right\}$$

where $$s_{n} = \left( {\beta_{12} \left( {k_{n}^{2} - \beta_{6} } \right) + \left( {\alpha_{10} k_{n}^{2} - \beta_{7} } \right)\left( {k_{n}^{2} - \beta_{11} } \right) - \left( {\alpha_{26} \left( {k_{n}^{2} - \beta_{6} } \right) - \left( {\alpha_{10} k_{n}^{2} - \beta_{7} } \right)\beta_{15} } \right)} \right),$$ $$\delta_{n} = \left( {\beta_{12} \left( {\beta_{5} k_{n}^{2} - \beta_{8} } \right) + \left( {\beta_{10} k_{n}^{2} - \beta_{14} } \right)\left( {\alpha_{10} k_{n}^{2} - \beta_{7} } \right)} \right),$$ $$d_{n} = \left( {\left( {\alpha_{26} - \alpha_{10} \beta_{15} } \right)k_{n}^{2} + \left( {\beta_{7} \beta_{15} - \beta_{6} \alpha_{26} } \right)} \right),$$$$f_{n} = \left( {\alpha_{27} \alpha_{10} k_{n}^{2} - \beta_{9} \alpha_{26} - \beta_{7} \alpha_{22} } \right),$$ $$m_{n} = \left( {\left( {\alpha_{26} \beta_{5} - \beta_{7} \alpha_{22} + \beta_{16} \alpha_{10} } \right)k_{n}^{2} + \alpha_{10} \alpha_{22} k_{n}^{4} - \beta_{8} \alpha_{26} } \right)\left( {\frac{{ - s_{n} }}{{\delta_{n} }}} \right),$$$$g_{n} = \alpha_{26} \left( {k_{n}^{2} - \beta_{6} } \right) - \beta_{9} \alpha_{26} H_{2n},$$ $$E_{n} = \alpha_{26} \left( {\beta_{5} k_{n}^{2} - \beta_{8} } \right)H_{1n},$$ $$p_{n} = \alpha_{26} \left( {\alpha_{10} k_{n}^{2} - \beta_{7} } \right),$$$$H_{1n} = \frac{{ - s_{n} }}{{\delta_{n} }},$$$$H_{2n} = \frac{{ - (d_{n} + m_{n} )}}{{f_{n} }},$$$$H_{3n} = \frac{{ - (g_{n} + E_{n} )}}{{p_{n} }},$$ $$n = 1,2,3,4$$.

## Boundary conditions

We assume certain assumptions in this section about boundary conditions applied to the plane surface at $x = 0$ are of the form:

1. The thermal condition made using a laser pulse that causes uniform heating can be shown as follows:
  38 $$\frac{\partial T}{{\partial x}} = \frac{{ - q_{o} t^{2} e^{{ - \frac{t}{{t_{p} }}}} }}{{16t_{p}^{2} }}$$
  where $t_{p}$ is the pulse heat flux’s duration, and $q_{o}$ is a constant.
2. The plasma condition can be expressed as
  39 $$\frac{\partial N}{{\partial x}} = \frac{c}{{D_{e} }}N$$
  Here c is a selected (positive) parameter, and $D_{e}$ is the diffusion coefficient of electron charge.
3. During the optical-excitation processes, the recombination diffusion for the hole charge field takes place at the surface $x = 0$, and may be written as
  40 $$H = h_{o}$$
4. The condition of mechanical ramp-type for the normal stress component at the surface $x = 0$ takes the form
  41 $$\sigma_{xx} = \left\{ {\begin{array}{*{20}l} {0,} \hfill & {\quad t \le 0,} \hfill \\ {\frac{{\text{t}}}{{t_{o} }},} \hfill & {\quad 0 < t \le t_{o} } \hfill \\ {1,} \hfill & {\quad t > t_{o} } \hfill \\ \end{array} } \right.$$
5. The shearing stress is traction-free at the surface $x= 0$, then
  42 $$\tau_{xz} = 0$$

Combining Eqs. (38)–(41) yields five equations for the constants *A*, ${L}_{1}$, ${L}_{2}$, ${L}_{3}$ and ${L}_{4}$.

43 $$\begin{gathered} k_{1} L_{1} + k_{2} L_{2} + k_{3} L_{3} + k_{4} L_{4} = R_{n} \hfill \\ k_{5} L_{1} - k_{6} L_{2} - k_{7} L_{3} - k_{8} L_{4} = 0 \hfill \\ H_{21} L_{1} + H_{22} L_{2} + H_{23} L_{3} + H_{24} L_{4} = R_{L} \hfill \\ R_{s1} L_{1} + R_{s2} L_{2} + R_{s3} L_{3} + R_{s4} L_{4} - R_{t} L_{5} = R_{m} \hfill \\ k_{9} L_{1} + k_{10} L_{2} + k_{11} L_{3} + k_{12} L_{4} - k_{13} L_{5} = 0 \hfill \\ \end{gathered}$$

where $${R}_{n}=\frac{{q}_{o}{t}^{2}{e}^{-\frac{t}{{t}_{p}}}}{16{{t}_{p}}^{2}}{e}^{-\left(\omega t+ibz\right)},{R}_{L}={h}_{o}{e}^{-\left(\omega t+ibz\right)},{k}_{5}=-{k}_{1}{H}_{31}-\frac{\text{c}}{{D}_{e}}{H}_{31},{k}_{6}={k}_{2}{H}_{32}+\frac{\text{c}}{{D}_{e}}{H}_{32},$$ $${k}_{7}={k}_{3}{H}_{33}+\frac{\text{c}}{{D}_{e}}{H}_{33},{k}_{8}={k}_{4}{H}_{34}+\frac{\text{c}}{{D}_{e}}{H}_{34},$$ $${k}_{9}=2ib\mu {k}_{1}{H}_{11},$$ $${k}_{10}=2ib\mu {k}_{2}{H}_{12},$$ $${k}_{11}=2ib{k}_{3}{H}_{13},$$ $${k}_{12}=2ib\mu {k}_{4}{H}_{14},$$ $${k}_{13}=({b}^{2}+\frac{{\upbeta }_{17}}{{\alpha }_{31}}),$$$${R}_{sn}={{k}_{n}}^{2}{H}_{1n}-{b}^{2}{\alpha }_{36}{H}_{1n}+{\alpha }_{37}{H}_{3n}+{\alpha }_{38}{H}_{2n}-(1+{\tau }_{\theta }\omega ),$$ $${R}_{t}=ib\sqrt{\frac{{\upbeta }_{17}}{{\alpha }_{31}}}+ib{\alpha }_{36}\sqrt{\frac{{\upbeta }_{17}}{{\alpha }_{31}}},$$ $${R}_{m}=\frac{\text{t}}{{t}_{o}}{e}^{-(\omega t+ibz), }$$ $${R}_{wn}=-{{b}^{2}H}_{1n}+{\alpha }_{36}{{k}_{n}}^{2}{H}_{1n}+{\alpha }_{37}{H}_{3n}+{\alpha }_{38}{H}_{2n}-(1+{\tau }_{\theta }\omega ),$$$${R}_{b}=ib\sqrt{\frac{{\upbeta }_{17}}{{\alpha }_{31}}}-ib{\alpha }_{36}\sqrt{\frac{{\upbeta }_{17}}{{\alpha }_{31}}},$$$$\text{n}=\text{1,2},\text{3,4}$$.

To calculate the constants ${L}_{1}$, ${L}_{2}$, ${L}_{3}$, ${L}_{4}$ and ${L}_{5}$, Cramer’s method is applied on Eqs. (43).

44 $$L_{1} = \frac{{\Delta L_{1} }}{\Delta },L_{2} = \frac{{\Delta L_{2} }}{\Delta },L_{3} = \frac{{\Delta L_{3} }}{\Delta },L_{4} = \frac{{\Delta L_{4} }}{\Delta },L_{5} = \frac{{\Delta L_{5} }}{\Delta }$$

The following dimensionless expressions of physical quantities (*T*, $N$, *H*, $u$, $w$,${\sigma }_{xx}$,${\sigma }_{zz}$, ${\tau }_{xz}$) can be derived from Eqs. (28)–(37) and (20)

45 $$T = \left\{ {\mathop \sum \limits_{n = 1}^{4} L_{n} e^{{ - k_{n} x}} } \right\}e^{{\left( {\omega t + ibz} \right)}}$$

46 $$N = \left\{ {\mathop \sum \limits_{n = 1}^{4} H_{3n} L_{n} e^{{ - k_{n} x}} } \right\}e^{{\left( {\omega t + ibz} \right)}}$$

47 $$H = \left\{ {\mathop \sum \limits_{n = 1}^{4} H_{2n} L_{n} e^{{ - k_{n} x}} } \right\}e^{{\left( {\omega t + ibz} \right)}}$$

48 $$u = - \left\{ {\mathop \sum \limits_{n = 1}^{4} k_{n} H_{1n} L_{n} e^{{ - k_{n} x}} + ibL_{5} e^{{ - \sqrt {\frac{{{\upbeta }_{17} }}{{\alpha_{31} }}} x}} } \right\}e^{{\left( {\omega t + ibz} \right)}}$$

49 $$w = \left\{ {\mathop \sum \limits_{n = 1}^{4} ibH_{1n} L_{n} e^{{ - k_{n} x}} - \sqrt {\frac{{{\upbeta }_{17} }}{{\alpha_{31} }}} L_{5} e^{{ - \sqrt {\frac{{{\upbeta }_{17} }}{{\alpha_{31} }}} x}} } \right\}e^{{\left( {\omega t + ibz} \right)}}$$

50 $$\sigma_{xx} = \left\{ {\mathop \sum \limits_{n = 1}^{4} R_{sn} L_{n} e^{{ - k_{n} x}} - R_{t} L_{5} e^{{ - \sqrt {\frac{{{\upbeta }_{17} }}{{\alpha_{31} }}} x}} } \right\}e^{{\left( {\omega t + ibz} \right)}}$$

51 $$\sigma_{zz} = \left\{ {\mathop \sum \limits_{n = 1}^{4} R_{wn} L_{n} e^{{ - k_{n} x}} - R_{b} L_{5} e^{{ - \sqrt {\frac{{{\upbeta }_{17} }}{{\alpha_{31} }}} x}} } \right\}e^{{\left( {\omega t + ibz} \right)}}$$

52 $$\tau _{{xz}} = \frac{{ - \mu }}{{\lambda + 2\mu }}\left\{ {\mathop \sum \limits_{{n = 1}}^{4} 2ibk_{n} H_{{1n}} L_{n} e^{{ - k_{n} x}} - k_{{13}} L_{5} e^{{ - \sqrt {\frac{{{{\upbeta }}_{{17}} }}{{\alpha _{{31}} }}} x}} } \right\}e^{{\left( {\omega t + ibz} \right)}}$$

## Numerical results and discussion

In this section, we delve into the fascinating world of wave propagation within a semiconductor medium. Our approach involves numerical simulations to study the main fields, including temperature, plasma (carrier density), thermal stress and holes charge carrier field distributions. To achieve this, we employ the physical constants specific to silicon (Si) material. These parameters listed in Table 1 are expressed in SI units and are meticulously. For a comprehensive understanding, we plan to utilize a MATHIMATICA program to graphically represent the fundamental distribution of these fields^19–21^.

**Table 1:** The physical constants for silicon (Si) material.

Unit	Symbol	Value	Unit	Symbol	Value
N ${\text{m}}^{ - 2}$	$\lambda$	$6.4 \times 10^{10}$	${\text{m}}^{2} \;{\text{s}}^{ - 1}$	$D_{h}$	$.125 \times 10^{ - 2}$
N ${\text{m}}^{ - 2}$	$\mu$	$6.5 \times 10^{10}$	${\text{m}}^{2} \;{\text{s}}^{ - 1}$	$\alpha_{n}$	$1 \times 10^{ - 2}$
${\text{kg}}\;{\text{m}}^{ - 3}$	$\rho$	2330	${\text{m}}^{2} \;{\text{s}}^{ - 1}$	$\alpha_{h}$	$5 \times 10^{ - 3}$
K	$T_{o}$	800	${\text{m}}^{2} \;{\text{s}}^{ - 1}$	$D_{e}$	$2.5 \times 10^{ - 3}$
Sec (s)	$\tau$	$5 \times 10^{ - 5}$	${\text{m}}^{ - 1}$	$\delta$	3
${\text{K}}^{ - 1}$	$\alpha_{t}$	$4.14 \times 10^{ - 6}$	${\text{J}}\;{\text{m}}^{ - 2}$	$p$	$10^{11}$
${\text{W}}\;{\text{m}}^{ - 1} \;{\text{K}}^{ - 1}$	$K$	150	${\text{Col}}^{2} \;{\text{cl}}^{ - 1} \;{\text{cm}}\;{\text{s}}$	$\sigma_{o}$	$9.36 \times 10^{5}$
${\text{J}}\;{\text{Kg}}^{ - 1} \;{\text{K}}^{ - 1}$	$C_{e}$	695	${\text{m}}^{ - 3}$	$n_{o}$	$10^{20}$
${\text{v}}\;{\text{K}}^{ - 1}$	$m_{qn}$	$1.4 \times 10^{ - 5}$	${\text{m}}^{ - 3}$	$h_{o}$	$10^{20}$
${\text{v}}\;{\text{K}}^{ - 1}$	$m_{nq}$	$1.4 \times 10^{ - 5}$	${\text{H}}\;{\text{m}}^{ - 1}$	$\mu_{e}$	$4\pi \times 10^{ - 7}$
${\text{v}}\;{\text{K}}^{ - 1}$	$m_{qh}$	$- .004 \times 10^{ - 6}$	PS	$t_{o}$	4
${\text{v}}\;{\text{K}}^{ - 1}$	$m_{hq}$	$- .004 \times 10^{ - 6}$	eV	$E_{g}$	1.11
${\text{m}}^{2} \;{\text{s}}^{ - 1}$	$D_{n}$	$.35 \times 10^{ - 2}$	Sec (s)	$\tau_{\theta }$	$5 \times 10^{ - 5}$

**Fig. 2:**
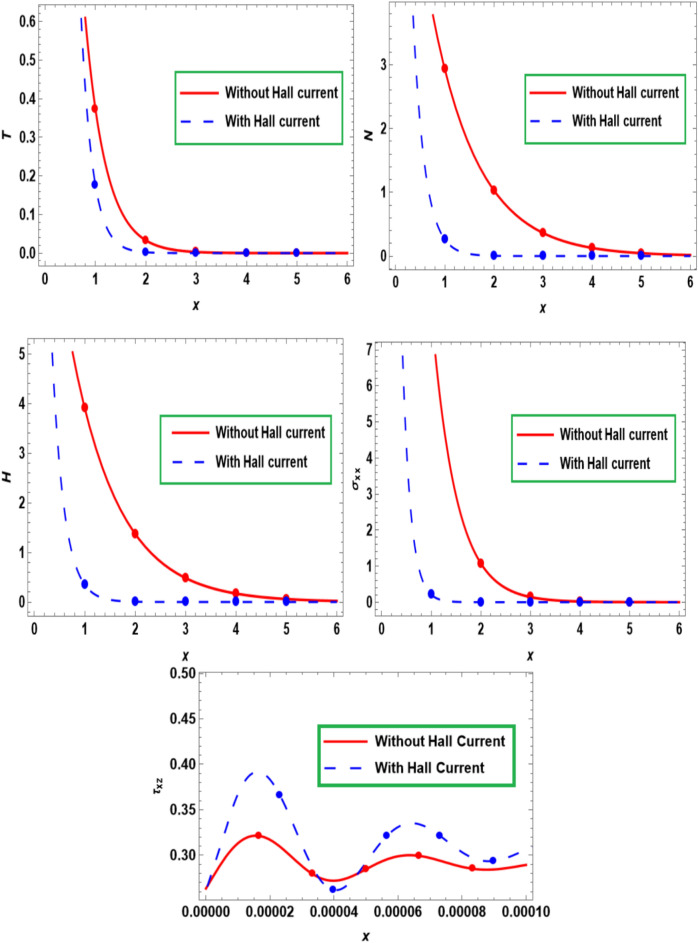
Influence of hall current on primary fields fluctuate with the distance $x.$

Figure 2 illustrates the deviation of the semiconducting medium in the main physical fields versus the dimensionless distance $x$ with and without strong magnetic field or hall current. It is clear that, there is a maximum variation in temperature $T$, carrier density $N,$ hole charge carrier concentration $H$ and thermal stress $\sigma_{xx}$ in the absence of hall effect. Also, variation in physical fields as ($T$, $N$, $H$, and $\sigma_{xx}$) are sharply decrease by rising the concentration of free electrons and holes (i.e. in the presence of $H$). But the opposite is happened with the tangential stress $\tau_{xz}$. Moreover, in the large values of $x$, there is no variation in these quantities $T,N,H,$ and $\sigma_{xx}$.

**Fig. 3:**
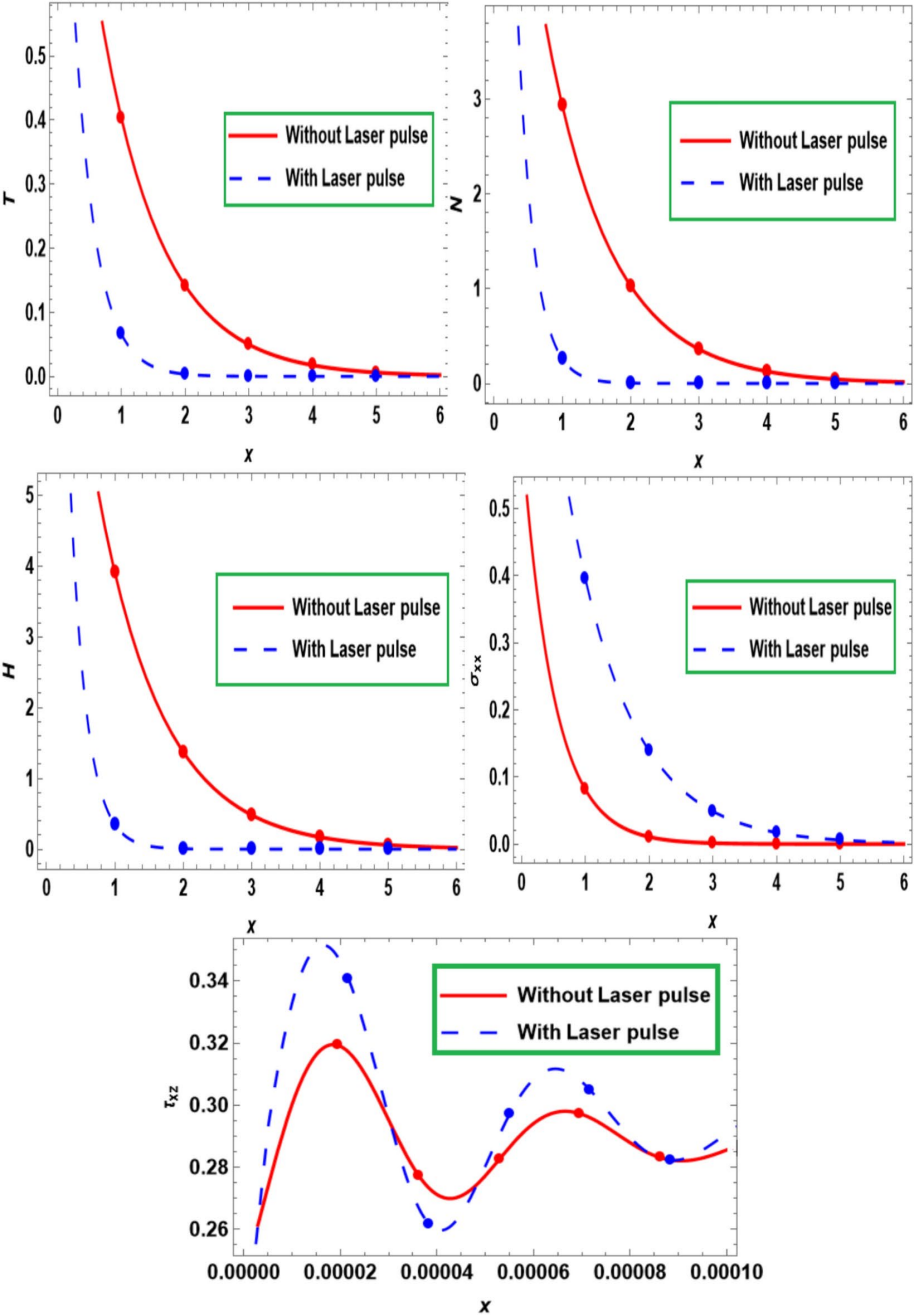
Influence of laser pulse on primary fields fluctuate with the distance $x.$

Figure 3 demonstrates the deviation of the wave propagation for the principal variables versus the dimensionless distance $x$ of the semiconducting medium with and without laser pulse effect. The magnitude of $T$, $N$, and $H$ reach to a maximum variation in the absence of laser pulse effect$.$ Additionally, presence of laser pulse effect causes the smaller variation in $T$, $N$, and $H$. In other words, $T$, $N$, and $H$ both experience a decrease in the range that follows an exponentially decreasing trend as the distance between them increases. Furthermore, with the thermal stresses, the amplitude has a maximum variation in the presence of a laser pulse but rapidly decreases in the absence of a laser pulse. This trend continues until the distributions are close to one another, at which point they converge to the zero line and are in agreement with the experimental findings^29^. However, when a strong magnetic field is paired with a Hall current, the semiconductor lattice’s interior particles are rearranged (along with the spindle movement of particles). By raising the concentration of holes and free electrons on the semiconductor’s surface, the Hall current boosts the flow of electric current within the material.

**Fig. 4:**
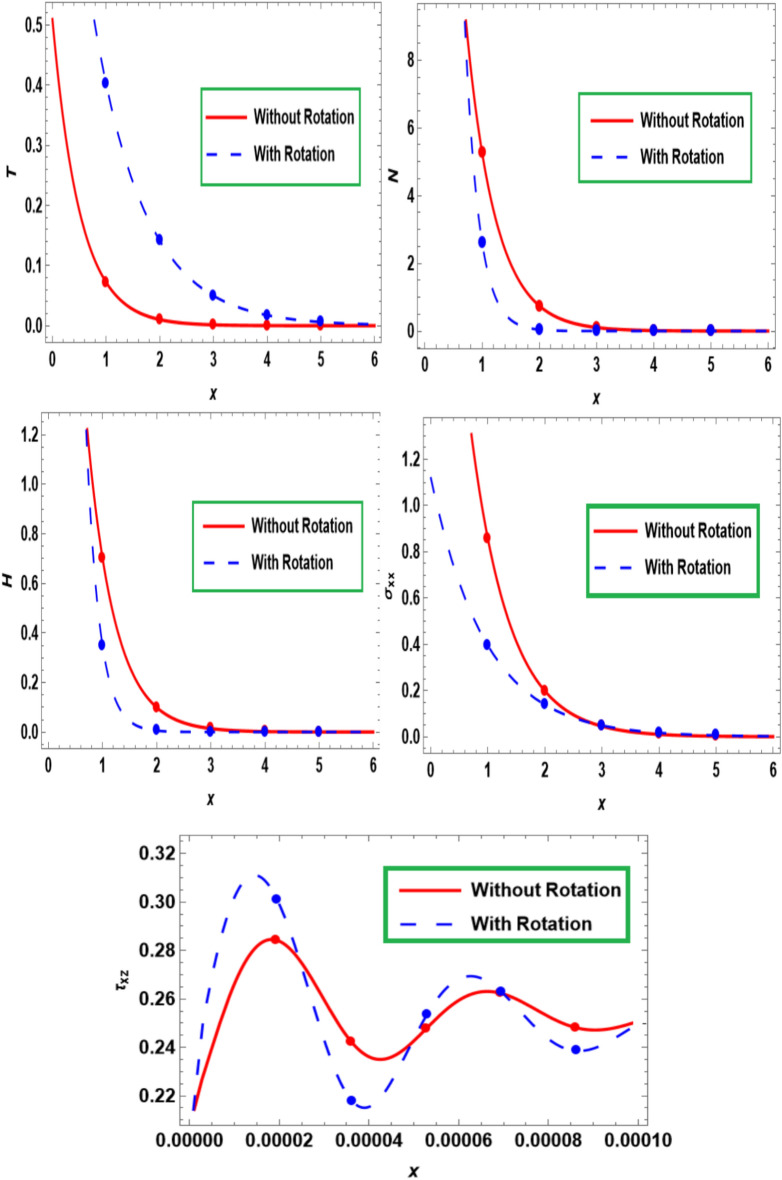
The primary fields fluctuate with the distance $x$ under the influence of rotation.

Figure 4 demonstrates the deviation in the temperature $\left( T \right)$, carrier density $\left( N \right)$, hole charge carrier concentration $H$ and thermal stress ($\sigma_{xx}$ and $\tau_{xz}$) versus the dimensionless distance $x$ of the semiconducting medium with and without rotation effect. This graph shows that, there is a maximum variation in $N$,$H$ and $\sigma_{xx}$ when the rotation effect is absent. Additionally, presence of rotation effect causes the smaller variation in $T$, and $\tau_{xz}$. In the absence of rotation, our results reduce to those obtained by Lotfy^19–21^. This trend begins to gradually slope away from the surface until its convergence to the zero line (state of equilibrium), which agrees with the experimental findings^24^. By increasing the number of holes and free electrons on the semiconductor’s surface, the laser pulse increases the flow of electric current within the semiconductor**.**

**Fig. 5:**
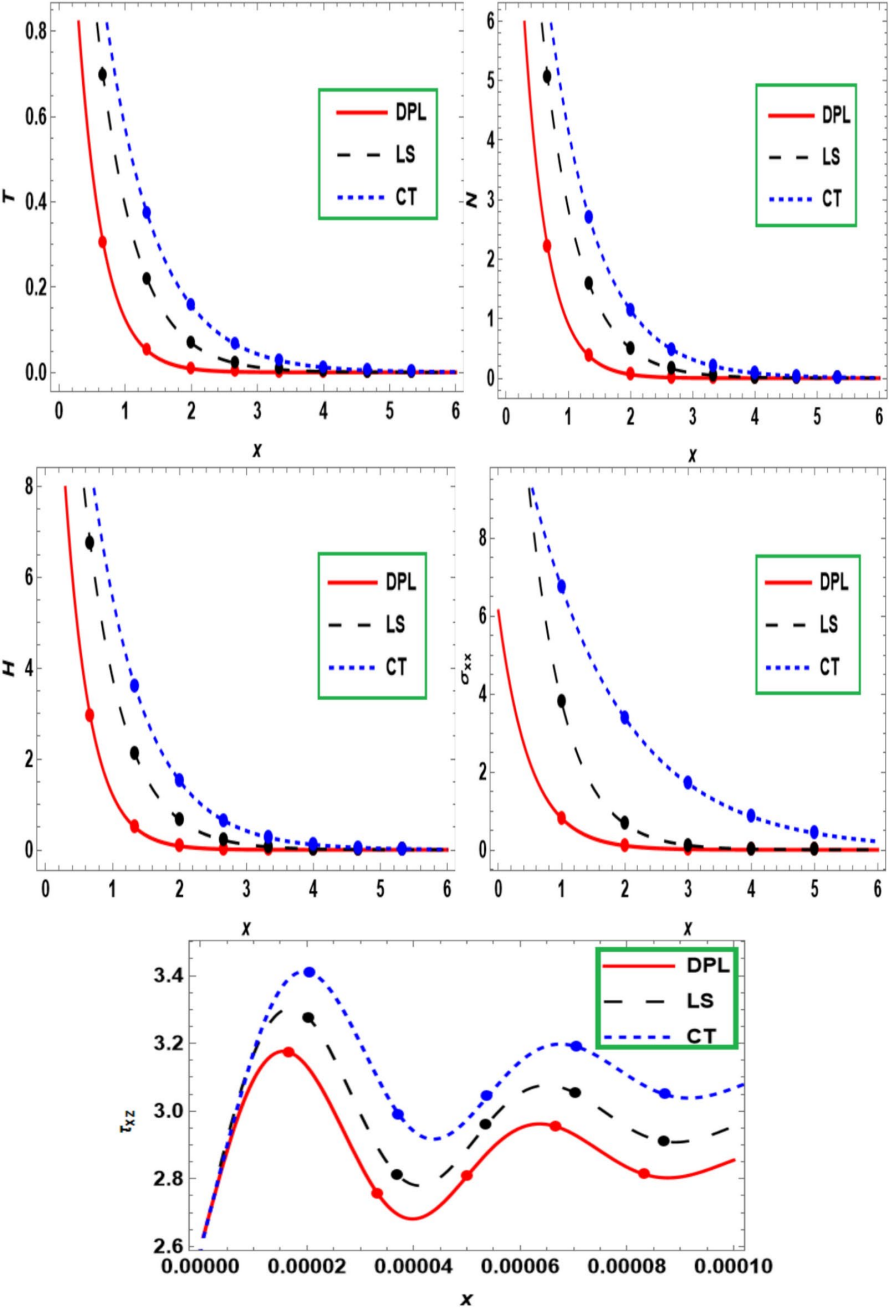
Influence of different thermoelasticity theories on main fields with the distance $x.$

Figure 5 displays the comparison between three models ($DPL$,${ }LS$ and $CT$) for the behavior of wave propagations of the main variables versus the dimensionless distance $x$. The semiconducting medium in the three models decreases gradually over the whole range. As rotation rises, the distribution of displacement decreases in models CT, G-L, and L-S but grows in model G-N, as the figures show. It has been noticed that the variation in $T,N,H,\sigma_{xx}$,$\tau_{xy}$ in $CT \succ LS \succ DPL.$ However, $DP L$ theory, $LS$ theory and $CT$ theory show the higher variation in different components.

**Fig. 6:**
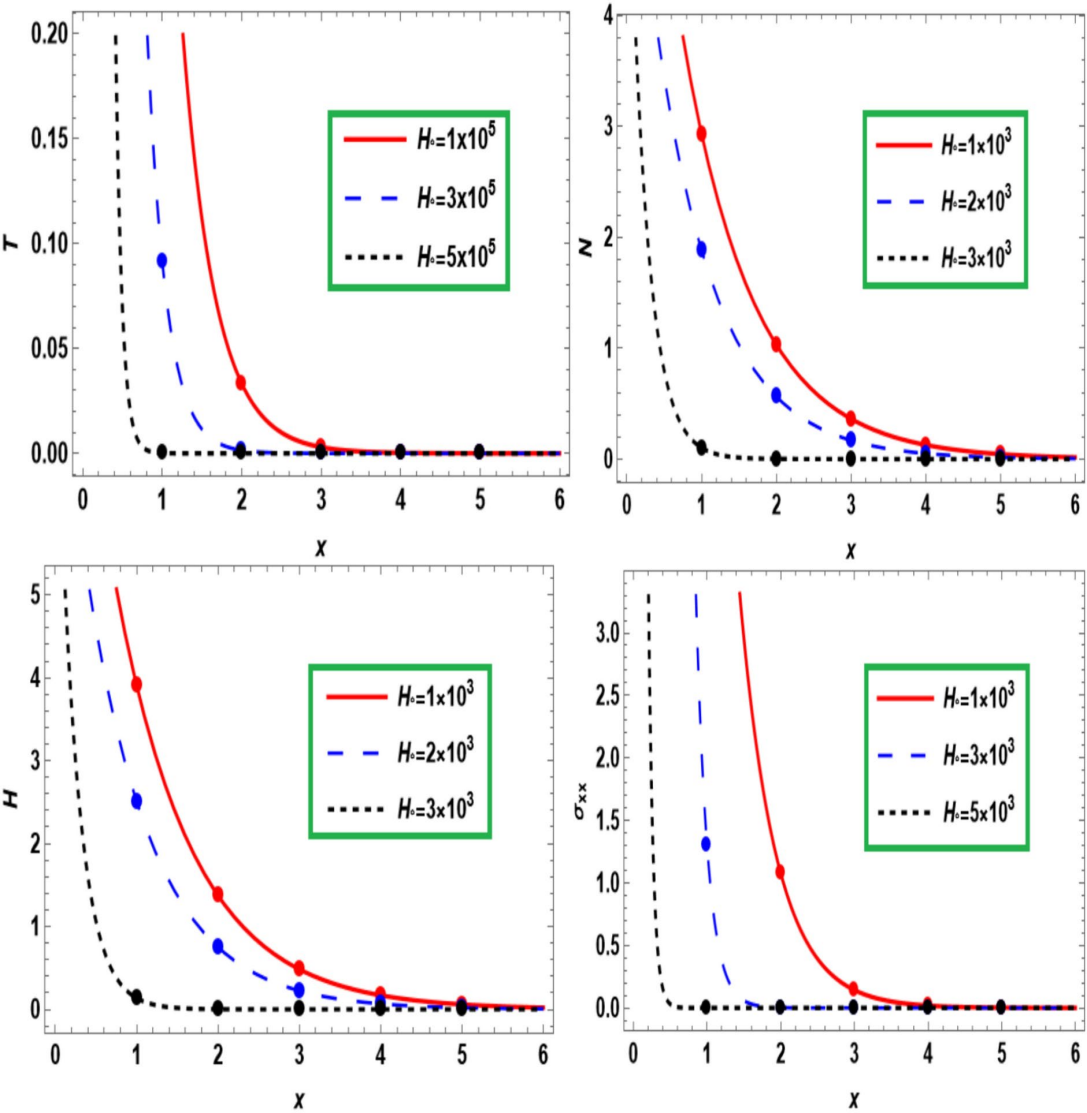
Influence of hall current on fundamental fields with the distance $x.$

Figure 6 shows how temperature (*T*), carrier density (*N*), hole charge carrier concentration (*H*), and normal stress ($\sigma_{xx}$) change with distance (*x*) in a semiconducting medium under the hall current effect. We discover that, the distribution of all quantities is reduced with increasing magnetic field. As *x* increases, all wave propagation diminishes and converges toward the zero line as the distance increases. This behavior seamlessly aligns with the expected characteristics of displacement components within semiconducture mediums, Hobiny et al.^31^, wherein higher harmonic vibrations invariably correlate with more rapid wave propagation.

**Fig. 7:**
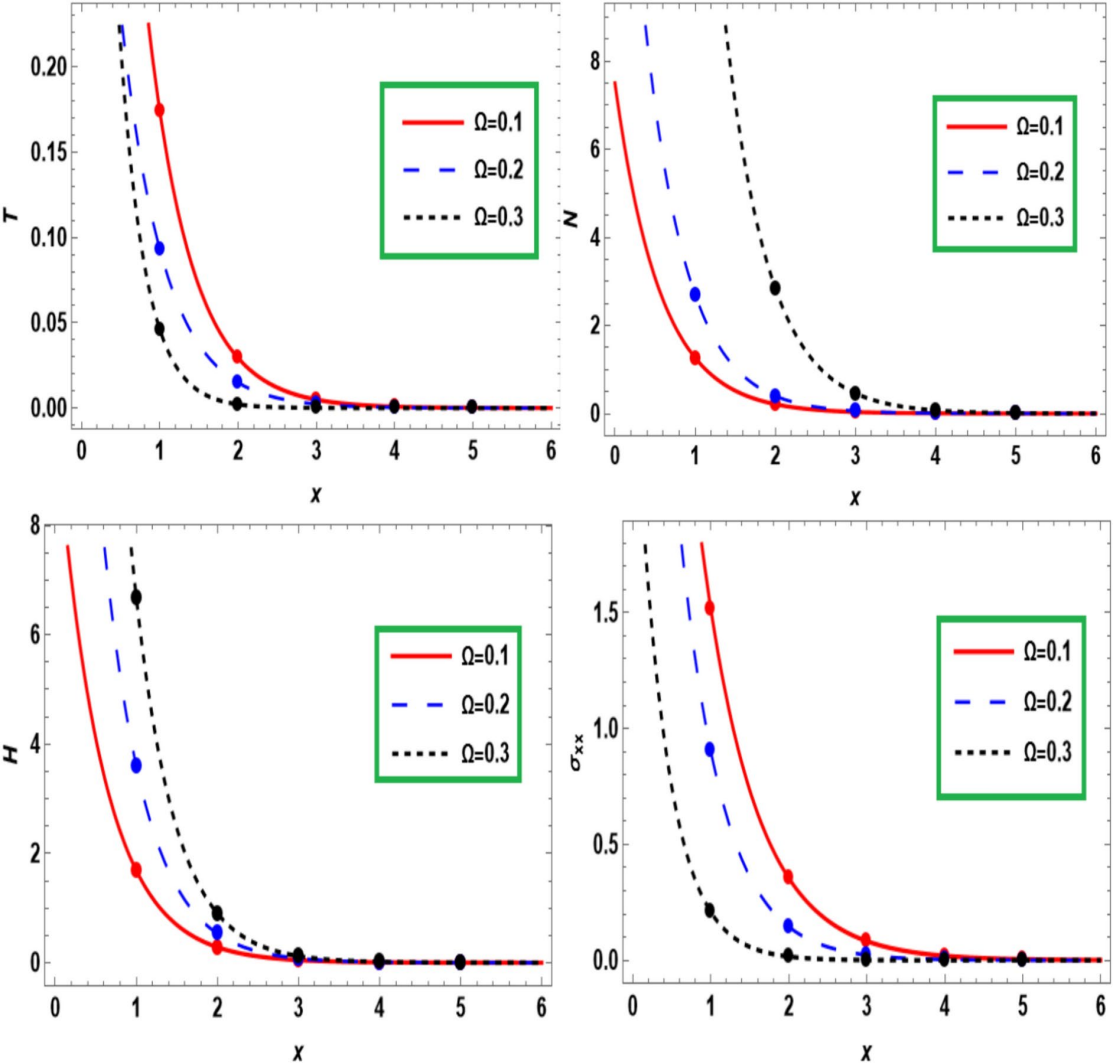
The rotation-effect on the fundamental fields at *x* distance.

Figure 7 represents the impact of rotation on the temperature $T$, carrier density $N,$ hole charge carrier concentration $H$ and normal stress $\sigma_{xx}$ versus the dimensionless distance $x$. From this figure, a significant difference in most physical fields (*T, N, H,* and $\sigma_{xx}$) is noticed for different values of rotation (${\Omega }$ = 0.1,${{ \Omega }}$ = 0.2,${{ \Omega }}$ = 0.3). The temperature $T$ and normal stress $\sigma_{xx}$ decrease with increasing values of rotation. But the profiles of holes charge carrier and carrier density (elastic) waves are observed to increase with increasing values of rotation. The propagation of all waves eventually decreases and converges to the zero line as the distance grows as in the steady state. Observing Fig. 7, it becomes apparent that the $N$ and $H$ rise with the rising value of rotation, while the $T$ and $\sigma_{xx}$ decrease with increasing of $\Omega$. Also, a sharp declination can be seen in the *T, N, H,* and $\sigma_{xx}$ with a higher distance extends regime due to rotation presence. In the case of half-space, the *T, N, H,* and $\sigma_{xx}$ falls sharply and accumulates into a single point in the higher regime of distance extends. According to experimental observations, the distribution decreases exponentially with distance from the surface until it reaches equilibrium at the zero line inside the semiconductor medium^29,30^.

**Fig. 8:**
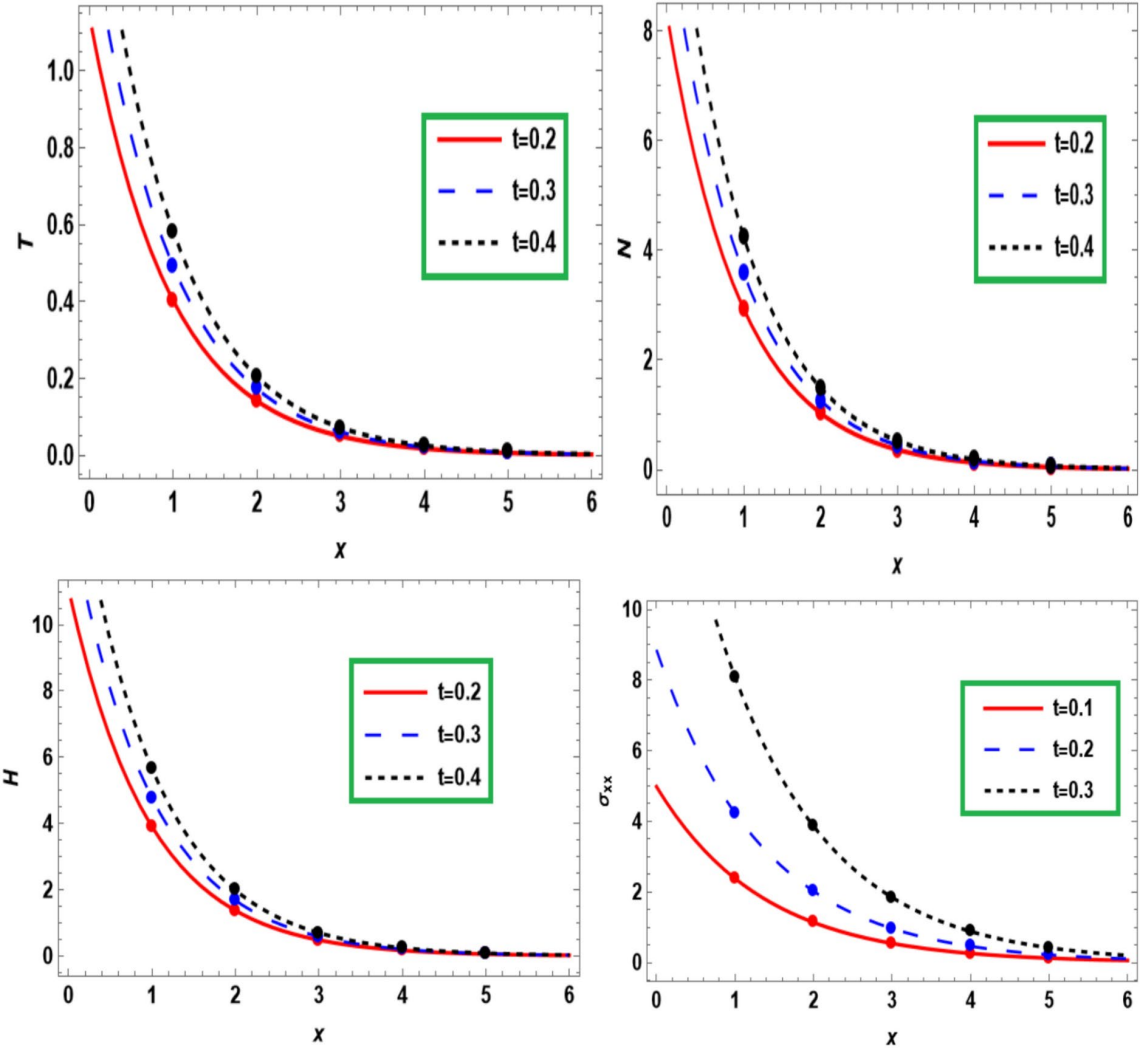
The time-effect on the fundamental fields at *x* distance.

Figure 8 examines the influence of time on the temperare $T$, carrier density $N,$ hole charge carrier concentration $H$ and normal stress $\sigma_{xx}$ versus the dimensionless distance $x$. It is clear that the time has a strong effect on all physical distributions, and it works to raise the amplitude of them as well as the waves decreases and converges to the zero line by increasing the distance and all lines snap together. In the case of greater distance values, the impact of time change on *T, N, H,* and $\sigma_{xx}$. is comparatively reduced. As distance extends to larger values, the variations in time have a diminishing impact on the overall *T, N, H,* and $\sigma_{xx}$. This behavior harmoniously aligns with the anticipated traits of normal stress and hole charge carrier concentration within the semiconductor medium described by Lotfy et al.^23^.

## Conclusion

This manuscript explored the impact of a powerful magnetic field and rotation on the behavior of photo-thermoelastic waves in semiconducting materials generated by laser pulses. Also, this study sheds light on the fascinating interconnections between magnetic fields, rotations, laser pulses, and semiconductor behavior, contributing to our understanding of fundamental physical processes. As well, the study of these models holds great importance for researchers in the field of photo-thermoelastic science as well as engineers. Furthermore, industrial applications can derive significant advantages, especially in the context of modern medical devices and sensors employed in electric cars. The investigation focused on several key aspects:

1. *Hole and electron interactions* By analyzing 2-D elastic and electronic deformation, the study revealed novel insights into the interactions between holes and electrons within the material.
2. *Photo-excited diffusion and optical energy* The significance of optical energy and photo-excited diffusion was thoroughly examined. These processes play a crucial role in the material’s response to external stimuli.
3. *Hall current generation* A Hall current occurred as a result of the intense magnetic field. The Hartmann number quantified this effect, providing valuable information about charge transport within the material. The Hall effect occurs when an electric current flow through a conductor (usually a semiconductor or metal) placed in a magnetic field perpendicular to the current direction. The Hall current is nonlinear and depends on the magnetic field strength and carrier concentration. The Hall coefficient changes with temperature, affecting sensor accuracy. The Hall effect helps determine material parameters like carrier density and conductivity. By studying the Hall effect, researchers gain insights into charge carrier mobility, concentration, and type (electrons or holes) in semiconductors.
4. *Hall effects and applications* Important information was obtained from the Hall current, which was produced by the magnetic field. Researchers learned a great deal about Hall potentiometers, linear Hall sensors, and semiconductors. These findings have broad applications across various scientific and technological domains, including automation, measurement, and electronics.
5. *Laser pulse impact* Surprisingly, few literature studies have explored the combined effects of Hall current on hole-electron interactions and the laser pulses. The study highlighted the intricate interplay between relaxation time and wave propagation. The model accounts for the thermal impact of laser rays, specifically non-Gaussian laser pulses, as they impinge upon the outer nonlocal surface of the material. This consideration is crucial for understanding the material’s response.
6. *Rotation impact* Rotation introduces a Doppler shift in the frequency of electromagnetic waves interacting with the semiconductor. This effect alters the effective energy levels of electrons and holes, influencing charge transport and optical properties. In practical terms, this can affect communication systems (such as optical fibers) and sensors that rely on precise frequency measurements. Rotation impacts semiconductor behavior across diverse apiary, plication’s, from fundamental physics to practical devices. So, engineers and researchers must consider rotational effects during design, optimization, and utilization of semiconductor-based systems.
7. As expected, it can be found that the shear stress satisfied the boundary condition.
8. The Hall current, a byproduct of the powerful magnetic field, also affects the wave propagation of the physical quantities of interest
9. *Modified physical variables* Laser pulses significantly modified the physical variables under investigation. The material behaved as predicted by science in the presence of a magnetic field, rotation and laser pulse.

## Data Availability

The datasets used and/or analyzed during the current study available from the corresponding author on reasonable request.

## Appendix 1

$$\begin{aligned} A_{11} = & \frac{ - 1}{\xi }\left\{ {\left( {\alpha_{10} \alpha_{27} \beta_{10} \beta_{18} - \alpha_{10} \alpha_{22} \beta_{13} \beta_{18} + \alpha_{10} \alpha_{16} \beta_{16} \beta_{18} + \alpha_{16} \alpha_{26} \beta_{19} + \alpha_{10} \alpha_{27} \beta_{19} - \alpha_{10} \alpha_{16} \beta_{15} \beta_{19} - \alpha_{10} \alpha_{16} \alpha_{22} \beta_{21} } \right.} \right. \\ & \quad + \alpha_{16} \alpha_{22} \beta_{22} + \alpha_{10} \alpha_{22} \beta_{23} + \alpha_{16} \alpha_{26} \beta_{18} \beta_{5} - \alpha_{16} \alpha_{22} \beta_{18} \beta_{7} \\ & \quad + \alpha_{2} \left( {\alpha_{10} \left( {\alpha_{22} \beta_{11} - \beta_{10} \beta_{15} - \beta_{16} } \right) + \alpha_{26} \left( {\beta_{10} - \beta_{5} } \right) + \alpha_{22} \left( { - \beta_{12} + \beta_{7} } \right)} \right) \\ & \quad + \alpha_{1} \left( {\alpha_{27} \left( { - \beta_{10} + \beta_{5} } \right) - \alpha_{16} \left( {\beta_{16} + \beta_{15} \beta_{5} } \right) + \alpha_{22} \left( {\beta_{13} + \alpha_{16} \beta_{6} + \beta_{9} } \right)} \right) \\ \end{aligned}$$

$$\begin{aligned} A_{{22}} = & \frac{1}{\xi }\left\{ {\left( {\alpha _{{10}} \alpha _{{27}} \beta _{{14}} \beta _{{18}} + \alpha _{{10}} \beta _{{13}} \beta _{{16}} \beta _{{18}} + \alpha _{{10}} \alpha _{{27}} \beta _{{11}} \beta _{{19}} - \alpha _{{27}} \beta _{{12}} \beta _{{19}} + \alpha _{{26}} \beta _{{13}} \beta _{{19}} } \right.} \right. \\ & \quad - \alpha _{{10}} \beta _{{13}} \beta _{{15}} \beta _{{19}} + \alpha _{{10}} \alpha _{{27}} \beta _{{10}} \beta _{{21}} - \alpha _{{10}} \alpha _{{22}} \beta _{{13}} \beta _{{21}} + \alpha _{{10}} \alpha _{{16}} \beta _{{16}} \beta _{{21}} \\ & \quad - \alpha _{{27}} \beta _{{10}} \beta _{{22}} + \alpha _{{22}} \beta _{{13}} \beta _{{22}} - \alpha _{{16}} \beta _{{16}} \beta _{{22}} + \alpha _{{26}} \beta _{{10}} \beta _{{23}} + \alpha _{{10}} \alpha _{{22}} \beta _{{11}} \beta _{{23}} \\ & \quad - \alpha _{{22}} \beta _{{12}} \beta _{{23}} - \alpha _{{10}} \beta _{{10}} \beta _{{15}} \beta _{{23}} - \alpha _{{10}} \beta _{{16}} \beta _{{23}} + \alpha _{{16}} \alpha _{{26}} \beta _{{24}} + \alpha _{{10}} \alpha _{{27}} \beta _{{24}} 3 \\ & \quad - \alpha _{{10}} \alpha _{{16}} \beta _{{15}} \beta _{{24}} - \alpha _{{27}} \beta _{{12}} \beta _{{18}} \beta _{5} + \alpha _{{26}} \beta _{{13}} \beta _{{18}} \beta _{5} + \alpha _{{16}} \alpha _{{26}} \beta _{{21}} \beta _{5} + \alpha _{{27}} \beta _{{22}} \beta _{5} \\ & \quad - \alpha _{{16}} \beta _{{15}} \beta _{{22}} \beta _{5} - \alpha _{{26}} \beta _{{23}} \beta _{5} + \alpha _{{16}} \alpha _{{26}} \beta _{{19}} \beta _{6} + \alpha _{{16}} \alpha _{{22}} \beta _{{22}} \beta _{6} + \alpha _{{27}} \beta _{{10}} \beta _{{18}} \beta _{7} \\ & \quad - \alpha _{{22}} \beta _{{13}} \beta _{{18}} \beta _{7} + \alpha _{{16}} \beta _{{16}} \beta _{{18}} \beta _{7} + \alpha _{{27}} \beta _{{19}} \beta _{7} - \alpha _{{16}} \beta _{{15}} \beta _{{19}} \beta _{7} - \alpha _{{16}} \alpha _{{22}} \beta _{{21}} \beta _{7} \\ & \quad + \alpha _{{22}} \beta _{{23}} \beta _{7} + \alpha _{{16}} \alpha _{{26}} \beta _{{18}} \beta _{8} - \alpha _{2} (\alpha _{{10}} \beta _{{14}} \beta _{{15}} + \alpha _{{10}} \beta _{{11}} \beta _{{16}} - \beta _{{12}} \beta _{{16}} - \beta _{{12}} \beta _{{15}} \beta _{5} \\ & \quad + \alpha _{{22}} \beta _{{12}} \beta _{6} - \alpha _{{22}} \beta _{{11}} \beta _{7} + \beta _{{10}} \beta _{{15}} \beta _{7} + \beta _{{16}} \beta _{7} + \alpha _{{26}} \left( { - \beta _{{14}} + \beta _{{11}} \beta _{5} - \beta _{{10}} \beta _{6} + \beta _{8} } \right)) \\ & \quad + \alpha _{{26}} \beta _{{10}} \beta _{{18}} \beta _{9} - \alpha _{{22}} \beta _{{12}} \beta _{{18}} \beta _{9} + \alpha _{{26}} \beta _{{19}} \beta _{9} + \alpha _{{22}} \beta _{{22}} \beta _{9} \\ & \quad - \alpha _{1} \left( {\alpha _{{16}} \beta _{{16}} \beta _{6} + \beta _{{13}} \left( {\beta _{{16}} + \beta _{{15}} \beta _{5} - \alpha _{{22}} \beta _{6} } \right) + \alpha _{{27}} \left( {\beta _{{14}} - \beta _{{11}} \beta _{5} + \beta _{{10}} \beta _{6} - \beta _{8} } \right)} \right. \\ & \quad \left. {\left. {\left. { + \alpha _{{16}} \beta _{{15}} \beta _{8} - \alpha _{{22}} \beta _{{11}} \beta _{9} + \beta _{{10}} \beta _{{15}} \beta _{9} + \beta _{{16}} \beta _{9} } \right)} \right)} \right\} \\ \end{aligned}$$

$$\begin{aligned} A_{{33}} = & \frac{1}{\xi }\left\{ {\left( {\beta _{{13}} \beta _{{16}} \beta _{{22}} - \alpha _{{26}} \beta _{{14}} \beta _{{23}} - \beta _{{12}} \beta _{{16}} \beta _{{23}} - \alpha _{{26}} \beta _{{13}} \beta _{{24}} } \right.} \right. + \alpha _{{10}} \left( { - \beta _{{13}} \beta _{{16}} \beta _{{21}} + \beta _{{14}} \beta _{{15}} \beta _{{23}} + \beta _{{11}} \beta _{{16}} \beta _{{23}} } \right. \\ & \quad \left. { + \beta _{{13}} \beta _{{15}} \beta _{{24}} - \alpha _{{27}} \left( {\beta _{{14}} \beta _{{21}} + \beta _{{11}} \beta _{{24}} } \right)} \right) - \alpha _{{26}} \beta _{{13}} \beta _{{21}} \beta _{5} + \beta _{{13}} \beta _{{15}} \beta _{{22}} \beta _{5} + \alpha _{{26}} \beta _{{11}} \beta _{{23}} \beta _{5} \\ & \quad - \beta _{{12}} \beta _{{15}} \beta _{{23}} \beta _{5} - \alpha _{2} \alpha _{{26}} \beta _{{14}} \beta _{6} - \alpha _{2} \beta _{{12}} \beta _{{16}} \beta _{6} + \alpha _{1} \beta _{{13}} \beta _{{16}} \beta _{6} - \alpha _{{26}} \beta _{{13}} \beta _{{19}} \beta _{6} \\ & \quad - \alpha _{{22}} \beta _{{13}} \beta _{{22}} \beta _{6} + \alpha _{{16}} \beta _{{16}} \beta _{{22}} \beta _{6} - \alpha _{{26}} \beta _{{10}} \beta _{{23}} \beta _{6} + \alpha _{{22}} \beta _{{12}} \beta _{{23}} \beta _{6} - \alpha _{{16}} \alpha _{{26}} \beta _{{24}} \beta _{6} \\ & \quad + \alpha _{2} \beta _{{14}} \beta _{{15}} \beta _{7} + \alpha _{2} \beta _{{11}} \beta _{{16}} \beta _{7} - \beta _{{13}} \beta _{{16}} \beta _{{18}} \beta _{7} + \beta _{{13}} \beta _{{15}} \beta _{{19}} \beta _{7} + \alpha _{{22}} \beta _{{13}} \beta _{{21}} \beta _{7} \\ & \quad - \alpha _{{16}} \beta _{{16}} \beta _{{21}} \beta _{7} - \alpha _{{22}} \beta _{{11}} \beta _{{23}} \beta _{7} + \beta _{{10}} \beta _{{15}} \beta _{{23}} \beta _{7} + \beta _{{16}} \beta _{{23}} \beta _{7} + \alpha _{{16}} \beta _{{15}} \beta _{{24}} \beta _{7} + \alpha _{2} \alpha _{{26}} \beta _{{11}} \beta _{8} \\ & \quad - \alpha _{2} \beta _{{12}} \beta _{{15}} \beta _{8} + \alpha _{1} \beta _{{13}} \beta _{{15}} \beta _{8} - \alpha _{{26}} \beta _{{13}} \beta _{{18}} \beta _{8} - \alpha _{{16}} \alpha _{{26}} \beta _{{21}} \beta _{8} + \alpha _{{16}} \beta _{{15}} \beta _{{22}} \beta _{8} + \alpha _{{26}} \beta _{{23}} \beta _{8} \\ & \quad + \alpha _{{27}} ( - \beta _{{11}} \beta _{{22}} \beta _{5} + \beta _{{10}} \beta _{{22}} \beta _{6} - \beta _{{11}} \beta _{{19}} \beta _{7} - \beta _{{10}} \beta _{{21}} \beta _{7} - \beta _{{24}} \beta _{7} + \beta _{{14}} \left( {\beta _{{22}} + \alpha _{{11}} \beta _{6} - \beta _{{18}} \beta _{7} } \right) \\ & \quad - \alpha _{1} \beta _{{11}} \beta _{8} - \beta _{{22}} \beta _{8} + \beta _{{12}} \left( {\beta _{{24}} + \beta _{{21}} \beta 5 + \beta 19\beta _{6} + \beta _{{18}} \beta _{8} } \right)) + \alpha _{1} \beta _{{14}} \beta _{{15}} \beta _{9} + \alpha _{1} \beta _{{11}} \beta _{{16}} \beta _{9} \\ & \quad - \alpha _{{26}} \beta _{{14}} \beta _{{18}} \beta _{9} - \beta _{{12}} \beta _{{16}} \beta _{{18}} \beta _{9} - \alpha _{{26}} \beta _{{11}} \beta _{{19}} \beta _{9} + \beta _{{12}} \beta _{{15}} \beta _{{19}} \beta _{9} - \alpha _{{26}} \beta _{{10}} \beta _{{21}} \beta _{9} \\ & \quad \left. {\left. { + \alpha _{{22}} \beta _{{12}} \beta _{{21}} \beta _{9} - \alpha _{{22}} \beta _{{11}} \beta _{{22}} \beta _{9} + \beta _{{10}} \beta _{{15}} \beta _{{22}} \beta _{9} + \beta _{{16}} \beta _{{22}} \beta _{9} - \alpha _{{26}} \beta _{{24}} \beta _{9} } \right)} \right\} \\ \end{aligned}$$

$$\begin{aligned} A_{{44}} = & \frac{1}{\xi }\left\{ {\left( { - \beta _{{13}} \beta _{{16}} \beta _{{22}} \beta _{6} + \alpha _{{26}} \beta _{{14}} \beta _{{23}} \beta _{6} + \beta _{{12}} \beta _{{16}} \beta _{{23}} \beta _{6} + \alpha _{{26}} \beta _{{13}} \beta _{{24}} \beta _{6} + \beta _{{13}} \beta _{{16}} \beta _{{21}} \beta _{7} } \right.} \right. \\ & \quad - \beta _{{14}} \beta _{{15}} \beta _{{23}} \beta _{7} - \beta _{{11}} \beta _{{16}} \beta _{{23}} \beta _{7} - \beta _{{13}} \beta _{{15}} \beta _{{24}} \beta _{7} + \alpha _{{26}} \beta _{{13}} \beta _{{21}} \beta _{8} \\ & \quad - \beta _{{13}} \beta _{{15}} \beta _{{22}} \beta _{8} - \alpha _{{26}} \beta _{{11}} \beta _{{23}} \beta _{8} + \beta _{{12}} \beta _{{15}} \beta _{{23}} \beta _{8} + \alpha _{{27}} ( - \beta _{{14}} \beta _{{22}} \beta _{6} - \beta _{{12}} \beta _{{24}} \beta _{6} \\ & \quad + \beta _{{14}} \beta _{{21}} \beta _{7} + \beta _{{11}} \beta _{{24}} \beta _{7} - \beta _{{12}} \beta _{{21}} \beta _{8} + \beta _{{11}} \beta _{{22}} \beta _{8} ) + \alpha _{{26}} \beta _{{14}} \beta _{{21}} \beta _{9} + \beta _{{12}} \beta _{{16}} \beta _{{21}} \beta _{9} \\ & \quad \left. {\left. { - \beta _{{14}} \beta _{{15}} \beta _{{22}} \beta _{9} - \beta _{{11}} \beta _{{16}} \beta _{{22}} \beta _{9} + \alpha _{{26}} \beta _{{11}} \beta _{{24}} \beta _{9} - \beta _{{12}} \beta _{{15}} \beta _{{24}} \beta _{9} } \right)} \right\} \\ \end{aligned}$$

## List of symbols

$\rho$: The material density
$$T_{^\circ }$$: Absolute temperature
$u,w$: The displacement components
$\phi ,\psi$: Two scalar functions
$c_{e}$: The specific heat
*N*: The carrier density
$$\lambda , \mu$$: Lame’s constants
$\gamma = \left( {3\lambda + 2\mu } \right)\alpha_{t}$: The volume coefficient of thermal expansion
$$n_{o}$$: Electrons concentration
$$h_{o}$$: Holes concentration
$t^{h} ,t^{n}$: The holes and electron’s relaxation times
$\alpha_{n} ,\alpha_{h}$: Thermo-diffusive parameters of electrons and holes
$\tau_{q} ,\tau_{\theta }$: The thermal and elastic relaxation times (phase-lag)
$\alpha_{t}$: The linear thermal expansion coefficient
$d_{n}$: The coefficients of electronic deformation
$d_{h}$: The coefficient of holes deformation
$\delta_{n} = \left( {3\lambda + 2\mu } \right)d_{n}$: The electrons elasto-diffusive parameter
$\delta_{h} = \left( {3\lambda + 2\mu } \right)d_{h}$: The holes elasto-diffusive parameter
$E_{g}$: The energy gap
$m_{nq} ,m_{qn} ,m_{hq} ,m_{qh}$: Peltier–Dufour–Seebeck–Soret-like constants
k: The thermal conductivity
$$I_{o}$$: Pulse parameter
$${\Omega }$$: Rotation parameter
$$t$$: The time
$D_{n} ,D_{h}$: The diffusion coefficient of the electrons and holes
$\sigma_{ij}$: The stress components
$a_{n} ,a_{h} ,a_{Qn} ,a_{Qh}$: The flux-like constants
$b$: The wave number
$\omega$: The complex frequency
